# *Streptococcus pneumoniae* and other bacterial nasopharyngeal colonization seven years post-introduction of 13-valent pneumococcal conjugate vaccine in South African children

**DOI:** 10.1016/j.ijid.2023.05.016

**Published:** 2023-09

**Authors:** Sarah L. Downs, Courtney P. Olwagen, Lara Van Der Merwe, Susan A. Nzenze, Marta C. Nunes, Shabir A. Madhi

**Affiliations:** 1 South Africa Medical Research Council Vaccines and Infectious Diseases Analytics Research Unit, University of the Witwatersrand, Faculty of Health Science, Johannesburg, South Africa; 2 Department of Science/ National Research Foundation: Vaccine Preventable Diseases, University of the Witwatersrand, Faculty of Health Science, Johannesburg, South Africa; 3 Division of Public Health Surveillance and Response, National Institute for Communicable Diseases of the National Health Laboratory Service, Johannesburg, South Africa; 4 Infectious Diseases and Oncology Research Institute, University of the Witwatersrand, Faculty of Health Science, Johannesburg, South Africa

**Keywords:** Pneumococcus, Pneumococcal conjugate vaccine, Colonization, Serotyping, 19F, Density

## Abstract

**Objectives:**

Pneumococcal conjugate vaccines (PCVs) reduce pneumococcal-associated disease by reducing vaccine-serotype (VT) acquisition in vaccinated children, thereby interrupting VT transmission. The 7-valent-PCV was introduced in the South African immunization program in 2009 (13-valent-PCV since 2011) using a 2+1 schedule (at 6, 14, and 40 weeks of age). We aimed to evaluate temporal changes in VT and non-vaccine-serotype (NVT) colonization after 9 years of childhood PCV immunization in South Africa.

**Methods:**

Nasopharyngeal swabs were collected from healthy children <60-month-old (n = 571) in 2018 (period-2) and compared with samples (n = 1135) collected during early PCV7-introduction (period-1, 2010-11) in an urban low-income setting (Soweto). Pneumococci were tested for using a multiplex quantitative-polymerase chain reaction serotyping reaction-set.

**Results:**

Overall pneumococcal colonization in period-2 (49.4%; 282/571) was 27.5% lower than period-1 (68.1%; 773/1135; adjusted odds ratio [aOR]: 0.66; 95% confidence interval [CI]: 0.54-0.88). Colonization by VT was reduced by 54.5% in period-2 (18.6%; 106/571) compared with period-1 (40.9%; 465/1135; aOR: 0.41; 95% CI: 0.3-0.56). Nevertheless, serotype 19F carriage prevalence was higher (8.1%; 46/571) in period-2 compared with period-1 (6.6%; 75/1135; aOR: 2.0; 95% CI: 1.09-3.56). NVT colonization prevalence was similar in period-2 and period-1 (37.8%; 216/571 and 42.4%; 481/1135).

**Conclusion:**

There remains a high residual prevalence of VT, particularly 19F, colonization nine years post-introduction of PCV in the South African childhood immunization program.

## Highlights

• Pneumococcal conjugate vaccines have modestly reduced circulating vaccine serotypes in Soweto, South Africa.
• There is a residual of vaccine serotypes.
• Colonization by vaccine-serotype 19F remains high.
• Co-colonization is higher in our setting than observed elsewhere.

## Background

*Streptococcus pneumoniae* remains a leading cause of morbidity and mortality, with the prevalence of nasopharyngeal colonization and burden of invasive pneumococcal disease (IPD) being highest in low-middle-income settings (LMIS) [1]. Routine immunization of children with pneumococcal conjugate vaccines (PCVs) has led to a reduction in vaccine-serotype (VT) IPD globally, including in South Africa [1,2]. The introduction of PCV in South Africa (along with HIV interventions) resulted in a rate reduction of 1277 IPD cases per 100 000 child-years comparing the pre- (2005–2008) and post-PCV introduction (2012-2013) periods [2]. Nevertheless, the overall prevalence of pneumococcal nasopharyngeal (NP) colonization has remained unchanged compared with prior to routine childhood immunization with PCV, due to replacement by non-vaccine serotypes (NVT) and residual colonization by some VT, especially in African countries [3], [4], [5], [6]. Similarly, the burden of IPD in the PCV immunization era is dominated by NVT and some residual disease from VT [7]. Therefore, surveillance of PCV impact on colonization at a community level can detect longer-term changes in the NP niche, and signal a risk of an increase in IPD by residual VT or replacement NVT [8].

Colonization by VT in South Africa was reduced by 62% [9] in an urban setting three years after the introduction of PCV7, and by 68% in a rural setting four years post-PCV7 introduction [3]. Only three studies in sub-Saharan Africa (Malawi [4], The Gambia [5], and Kenya [6]) have assessed NP carriage in healthy children, more than five years after the implementation of PCVs. In addition, Malawi, The Gambia, and Kenya use a ‘3+0’ PCV dosing schedule, administering PCV at 6, 10, and 14 weeks of age without a booster dose [4], [5], [6]. South Africa differs from other sub-Saharan African countries and uses a ‘2+1’ schedule involving two priming doses administered at 6 and 14 weeks of age and a booster (40 weeks of age). The booster dose is key for the durability of protection against VT acquisition, disease, and transmission [10,11]. Waning mucosal immunity in settings using primary series only has limited the impact of PCV on reducing colonization and transmission [4,10,11]. Furthermore, studies in South Africa, The Gambia, Kenya, and Malawi investigated colonization in NP samples using culture-based methods, which hamper the detection of co-colonization. Real-time polymerase chain reaction (PCR) can be optimized to quantify both concurrent and lower-density colonization by VT, NVT, and other bacterial species [12].

Studies investigating the long-term impact of PCV ‘21’ in South Africa using comprehensive and sensitive typing schemes are warranted to monitor changes in pneumococcal serotype epidemiology and vaccine coverage in a setting with a mature PCV immunization program [13]. This will be important as South Africa considers transitioning to a reduced dosing schedule that involves one primary dose and one booster (‘1+1’) in order to alleviate the high costs of PCV [14]. Further, community-based surveillance could serve as a benchmark against which future changes in pneumococcal colonization profiles can be compared if childhood immunization schedules transition to PCV 1+1 doses.

The aim of this study was to evaluate the temporal changes of serotype-specific pneumococcal and other bacteria colonization in the nasopharynx of children ≤5 years of age, 9 years after the routine implementation of PCV (7 years after PCV13) into the childhood immunization program in South Africa.

## Methods

### Study population

In South Africa, routine PCV immunization of children at 6, 14, and 40 weeks (2+1 schedule) commenced in April 2009 (7-valent PCV [PCV7]) and transitioned to 13-valent PCV (PCV13) in May 2011 [9]. Serotype-specific colonization was evaluated in the early PCV7-era (May 2010 to February 2011; period-1) among children living with HIV (CLWH) and HIV-uninfected children, as described [9]. Briefly, children were enrolled in Soweto, at HIV treatment clinics within Chris Hani Baragwanath Academic Hospital, and through community state clinics. For the current analysis, archived nasopharyngeal (NP) swabs (aluminum-shafted, Dacron swab, Medical Wire and Equipment Co. Ltd., Corsham, Wiltshire, England) collected from children <60 months of age and stored in skim milk-tryptone-glucose-glycerol (STGG) transport media at -70°C, were retrospectively analyzed (period-1) and served as baseline for comparison. Samples collected from period-1 had previously been investigated by standard culture methods and may have undergone multiple (up to four) freeze-thaw cycles before this study [9]. Here, we used a comprehensive quantitative real-time PCR (qPCR) that is capable of detecting pneumococcus and other NP bacterial colonizers with high sensitivity and specificity [12].

To assess the changes in pneumococcal colonization, a further prospective carriage survey was undertaken in Soweto from June to December 2018 (period-2). In period-2, healthy children <60 months of age were enrolled through household-level surveys, selected using the World Health Organization (WHO) EPI (Expanded Program on Immunization) probability proportional to size cluster survey method detailed in the supplementary text. All healthy children <60 months of age within the selected households were sampled, with parental consent.

### Sample collection

In period-2, the nasopharynx of children <60 months of age were sampled using FLOQSwabs (Copan Diagnostics, Murrieta, CA, USA) and placed in 1.5 ml of STGG, on ice and transferred to the Vaccines, and Infectious Diseases Analytics Research Unit (Wits-VIDA) within 6 hours of collection. Samples (NP swabs) were then stored at -70°C, according to WHO recommendations [15]. On the day of NP sampling, information was collected about the child's health, and other co-variates, detailed in supplementary text.

### Total nucleic acid extraction and quantitative real-time polymerase chain reaction

Stored NP samples were thawed and vortexed (30 seconds). Nucleic acids were extracted using the NucliSens® easyMAG® extraction system (BioMérieux, Marcy l'Etoile, France) according to the manufacturer's instructions and stored at –20°C until tested. Nucleic acid extracts were tested using the Standard BioTools™ nanofluidic qPCR on the Biomark HD System within the 96.96 Dynamic Array integrated fluidic circuit (96.96 IFC) as described previously [12]. The qPCR could detect 92 pneumococcal serotypes and differentiate these into 35 serotypes in 16 groups, 57 individual types, and identify 15 other bacterial species.

### Statistical analysis

Statistical analysis was performed with STATA Version 13.1 (StataCorp, Texas, USA). The cycle of quantification (Cq) values were converted to density (Log_10_ genomic equivalents [GE]/ml), and the relevant algorithms were applied to interpret pneumococcal serotypes and bacterial colonizers, detailed previously [12]. Samples were considered positive for a target if the density was above the limit of detection for that assay set [16] and were considered positive for pneumococcus if at least three out of four included pneumococcal reference genes (Lyt*A*, Pia*B, Ply,* and Xisco) were detected. Samples positive for pneumococcus but without a designated serotype, were interpreted as non-typeable (NT) *S. pneumoniae*. Further, we compared the difference in the average density of Lyt*A* and Pia*B* and the cumulative density of detected serotypes using Bland Altman (BAT) plots. If the average Lyt*A* and Pia*B* density exceeded the upper 95% limit of agreement of the BAT plot, these were considered co-colonizing NT pneumococci. Similarly, where *Haemophilus influenzae* was detected using the IgA1 gene, but the capsular loci (Bex*A* and Bex*B*) and the type-b capsule were not detected, these were designated as NT *H. influenzae* (NTHi).

PCV immunization coverage was assessed in period-2 only, due to differences in the data collection methods. Children were categorized into age groups (6-14; >14-40; and >40 weeks of age) according to the South African PCV immunization schedule. The demographic characteristics were compared between the two periods using the Student's *t*-test for continuous variables or the Pearson Χ^2^ test for categorical variables. The multivariate analysis included *P*-values <0.1.

Multivariate logistic regression models were used to assess the temporal association of colonization prevalence of pneumococcal (overall, VT, and NVT) and other bacteria NP colonization in period-2 compared with period-1. We calculated adjusted odd ratios (aOR), adjusting for factors known to impact colonization including if the child was ever breastfed, HIV infection, antimicrobial use, co-trimoxazole prophylaxis, and tuberculosis treatment. We did not stratify children according to their HIV status, as the number of CLWH was expected to be low in period-2. When aOR could not be calculated, Fisher's exact test was used to test for statistical significance. Regression analysis was used to calculate the difference between the geometric mean density (GMD) in period-2 and period-1. Further, we used density to rank co-colonizers (serotypes or bacterial targets). To explore the inter-relationship between co-colonizers, we constructed a colonization hierarchy matrix based on a relative target density. We considered the highest-density colonizer as the primary colonizer and where lower-density colonizers were present, these were classified as secondary colonizers. The matrix was translated into paired relationships and chord diagrams were constructed with flourish.studio (available at https://flourish.studio). Logistic regression was used to compare co-colonization events. We did not adjust for multiple comparisons as this is a descriptive study, and the analysis was pre-planned. Nevertheless, to account for multiplicity, we considered *P-*values of ≤0.01 as significant.

## Results

A total of 1135 NP swabs collected during period-1 and 571 during period-2 were available for qPCR analysis. Overall, children were of a similar mean age (25.9 months) in period-1 and period-2, however, those <24 months and >24 months of age were 1 month younger and approximately 4 months older, respectively, in period-1, Table 1. Since some children were enrolled through HIV clinics in period-1, there were more CLWH and children taking co-trimoxazole prophylaxis in period-1 (47.4% and 22.6%) compared with period-2 (0.5%; *P* <0.001 and 0.4%; *P* <0.001), and more children were on tuberculosis treatment in period-1 (4%) compared with period-2 (0.4%; *P* <0.001). More children attended day-care in Period-1 (36.1%) compared with period-2 (20.1%; *P* <0.001), and the proportion of children who have ever breastfed was lower in period-1 (59%) compared with period-2 (79.6%; *P* <0.001). Since antibiotic usage was not an exclusion criterion for enrollment in period-1, 9.3% of the enrolled children used antibiotics within three weeks of sample collection. The coverage of three doses of PCV for children >40 weeks of age was 93.2% in 2018 (period-2), and 99.4% of the enrolled children had received at least one dose of PCV, Supplementary Table 1. The WHO/UNICEF Estimates of National Immunization Coverage (WUENIC) for PCV was 58% in 2010 for South Africa (https://immunizationdata.who.int/pages/coverage/PCV.html). The WUENIC estimates per year since 2009 are included in Supplementary Figure 1.

**Table 1:** Baseline characteristics of children 0-60 months of age enrolled in two cross-sectional surveys undertaken in Soweto, South Africa.

	Period 1 (2010; N = 1135)	Period 2 (2018; N = 571)	*P*-value
0-60 months: mean age in months (SD)	25.9 (17.8)	25.9 (16.0)	0.934
≤ 24 months: mean age in months (SD)	11.2 (6.0); N=616	12.3 (6.6); N=289	0.009
> 24 months: mean age in months (SD)	43.4 (9.3); N=519	39.7 (9.8); N=282	<0.001
Currently breastfed. n/N (%)	310/1134 (27.3)	137 (24%)	0.14
Ever breastfed n/N (%)	521/882 (59)	454 (79.5)	<0.001
Attendance at daycare n/N (%)	409/1134 (36.1)	115 (20.1)	<0.001
Children living with HIV n/N (%)	538 (47.4)	3 (0.5)	<0.001
Co-trimoxazole prophylaxis. n/N (%)	257 (22.6)	2 (0.4)	<0.001
Treated for Tuberculosis in the past year n/N (%)	45/1124 (4)	2 (0.4)	<0.001
Current antibiotic treatment n (%)	105 (9.3)	1 (0.2)	<0.001
Hospitalized in last 3 months n (%)	52 (4.6)	0	<0.001

Student's *t*-test or Pearson X^2^ test was used to compare demographic and risk factors for colonization between the cohorts and those with *P*-values <0.01 and known to affect pneumococcal colonization were included in multivariate analyses; n: number of individuals with investigated outcomes; N: total number of individuals with available information on the characteristic. N was only included in rows where N was different from that indicated in the column header.

### Pneumococcal colonization prevalence

**Figure 1:**
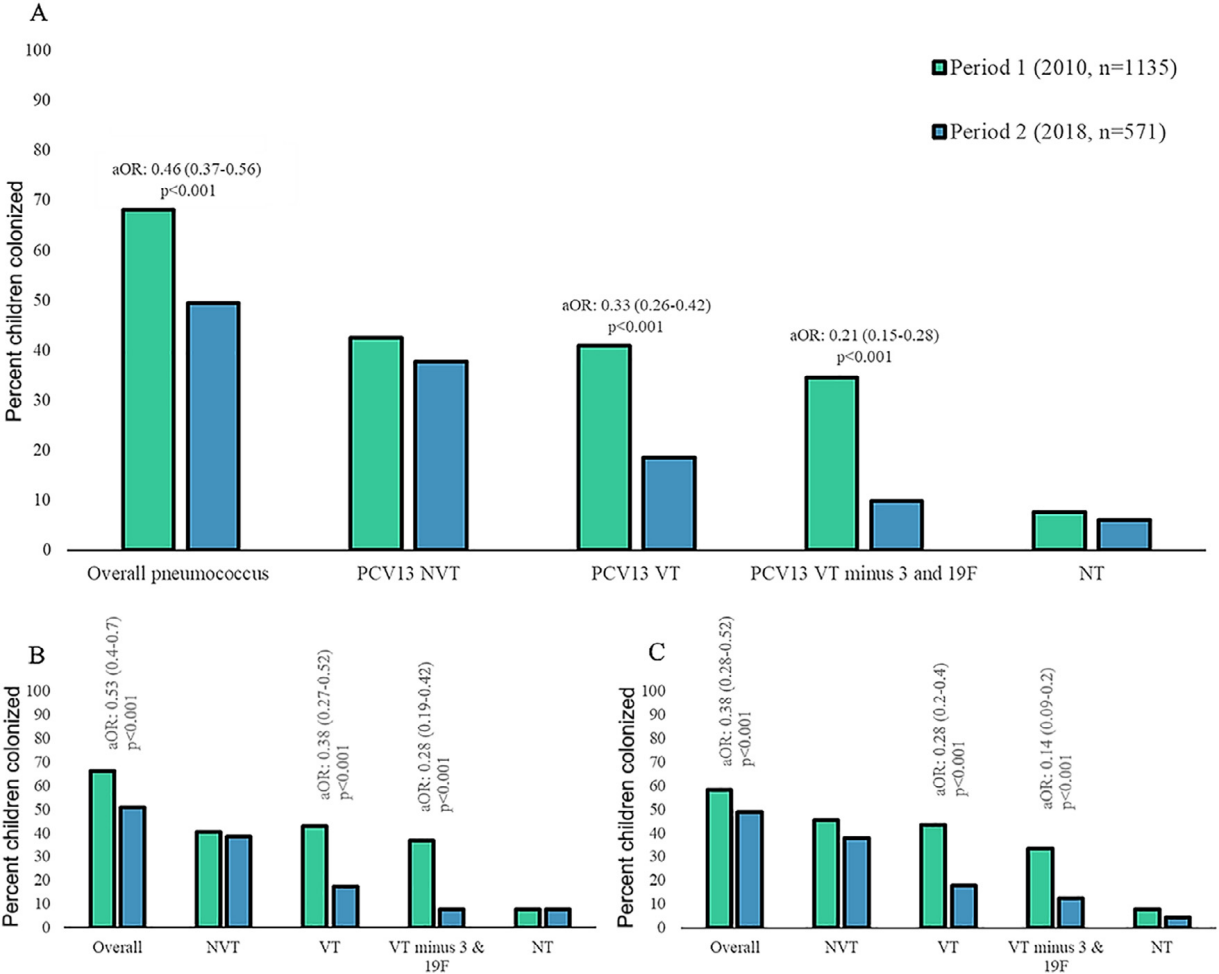
Colonization prevalence of *Streptococcus pneumoniae* in children aged 0-60 months. Panel A includes all children 0-60 months-of-age, Panel B includes children 0-24 months of age, and Panel C includes children 25-60 months of age. PCV13 NVT; PCV13 VT (1, 3, 4, 5,6A, 6B, 7A/F, 9A/V, 14, 18C, 19A, 19F, and 23F); NT *S. pneumoniae. P*-values <0.01 were considered significant, and calculated using logistic regression. In Panel B–C significant values are indicated by * and are detailed in Supplementary Table 2. aOR, adjusted odds ratio; NT, non-typeable; NVT, non-vaccine serotypes; VT, vaccine serotypes.

**Figure 2:**
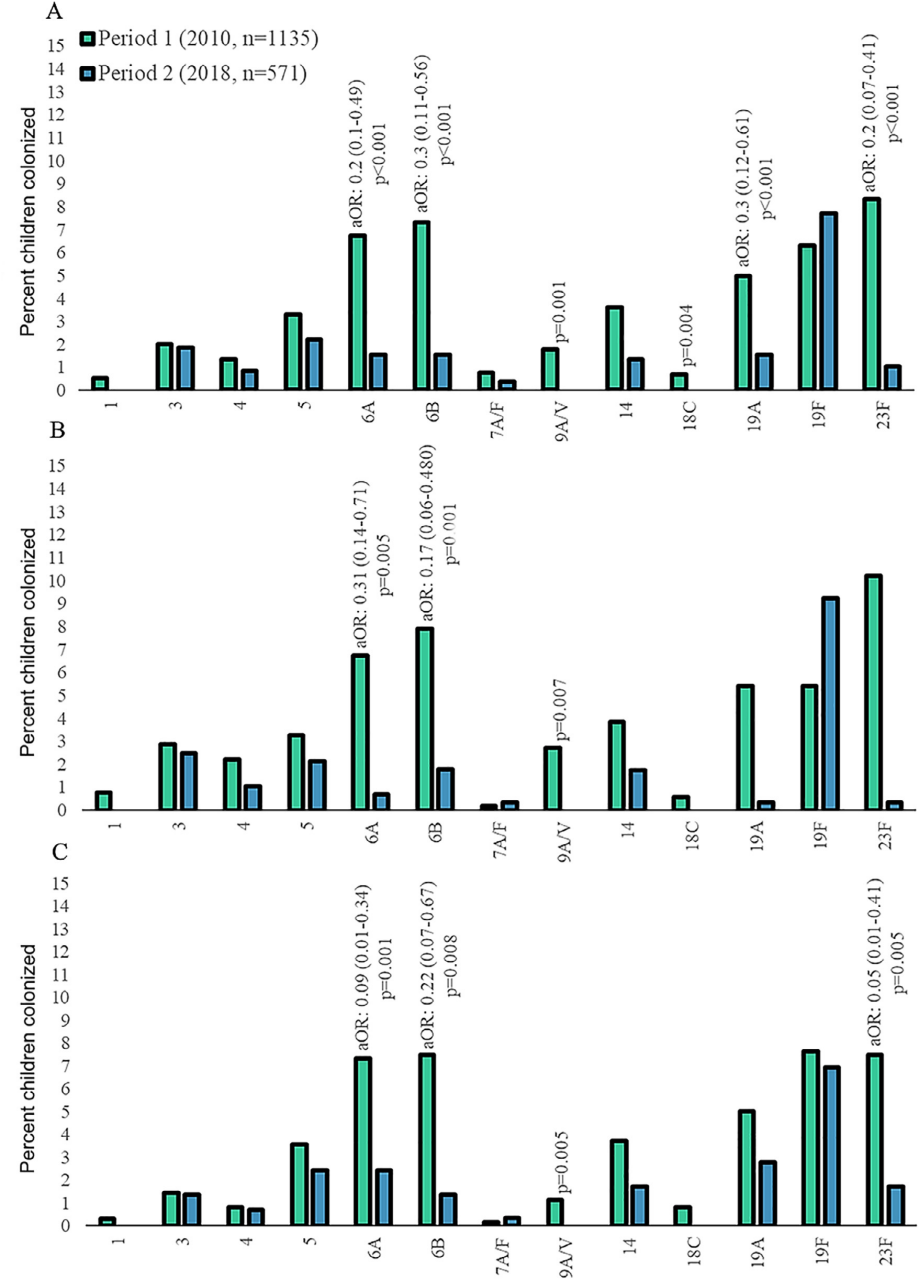
Colonization prevalence of pneumococcal vaccine serotypes in children 0-60 months of age. Panel A includes all children 0-60 months of age, Panel B includes children 0-24 months of age, and Panel C includes children 25-60 months of age. Only significant *P*-values shown, *P* <0.01 were considered significant, calculated using logistic regression. All other *P*-values are presented in Supplementary Table 2. aOR, adjusted odds ratio.

The overall prevalence of pneumococcal colonization was lower in period-2 (49.4%; 282/571) compared with period-1 (68.1%; 773/1135; aOR: 0.66; 95% confidence intervals [CI]: 0.54-0.88); Figure 1, Supplementary Table 2). This was driven by the 59% lower VT prevalence in period-2 (18.6%; 106/571) compared with period-1 (40.9%; 465/1135; aOR: 0.41; 95% CI: 0.3-0.56). The net reduction in VT colonization in period-2 compared with period-1 was due to a lower prevalence of colonization by serotypes 6A; 6B; 19A; and 23F (Figure 2, Supplementary Table 2). Nevertheless, the prevalence of serotype 19F was unchanged in period-2 (8.1%; 46/571) compared with period-1 (6.6%; 75/1135; aOR: 2.0; 95% CI: 1.09-3.56). The prevalence of NVT colonization was similar in period-2 (37.8%; 216/571) and period-1 (42.4%; 481/1135); Figure 1; Supplementary Table 2 and Supplementary Figure 2.

### Density of pneumococcal carriage

There was an increase in the overall pneumococcal GMD in the NP samples in period-2 (4.50 log_10_ GE/ml) compared with period-1 (4.32 log_10_ GE/ml; *P* = 0.01) Supplementary Figure 3; Supplementary Table 3. The GMD of VT was, however, lower in period-2 (3.92 log_10_ GE/ml) compared with period-1 (4.32 log_10_ GE/ml; *P* <0.001). Specifically, the GMD of VT 6A and 6B were lower in period-2 compared with period-1 (*P* <0.001 and *P* = 0.002, respectively); whereas 19A and 23F were higher (*P* = 0.003 and *P* = 0.005, respectively), and the other VT were unchanged; Supplementary Figure 4, Supplementary Table 3. The GMD of NVT were similar between periods (*P* = 0.089), Supplementary Figure 3; Supplementary Table 3, however the GMD 15A/F and 35B increased in period-2 compared with period-1 (*P* = 0.008 and *P* = 0.01, respectively; Supplementary Figure 5, Supplementary Table 3).

### Co-colonization of pneumococcal serotypes

There was no statistically significant difference in the number of individual circulating serotypes/serogroups between period-2 (n = 59) and period-1 (n = 63) or the percentage of children co-colonized (*P* = 0.48). In period-1 and period-2, 40.6% (314/773) and 37.9% (107/282) of children carried two or more, 13.5% (104/773) and 15.6% (44/282) at least three, and 4.9% (38/773) and 6.4% (18/282) carried at least four serotypes concurrently, respectively.

In co-colonized children, the detection of VT as the dominant colonizer (highest density or only) as a proportion of all the times VT was detected, was lower in period-2 (73.6%; 78/106) compared with period-1 (84.3%; 391/464; OR: 0.52; 95% CI: 0.32-0.86), whereas a higher proportion of NVT were identified as dominant colonizers in period-2 (87.0%; 180/207) compared with period-1 (70.1%; 331/472; OR: 2.84; 95% CI: 1.81-4.45).

**Figure 3:**
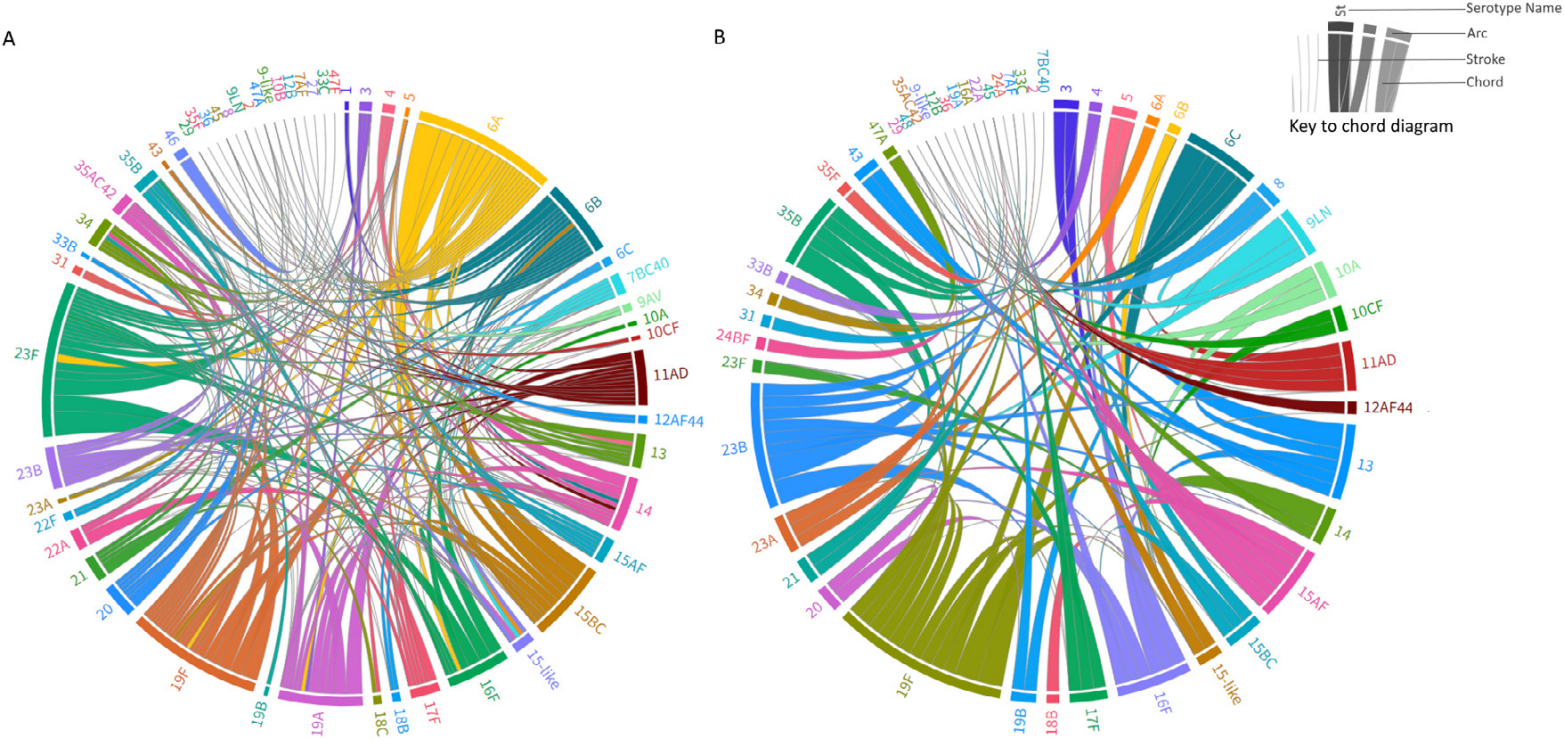
Chord diagram of co-colonizing events by pneumococcal serotypes in children 0-60 months of age in period-1 and period-2. Panel A includes period-1 (2010), and Panel B includes period-2 (2018). The right inset describes features of the diagram. Colors are assigned randomly. The outer circle contains arcs that represent each serotype. Each arc is paired with a co-colonizing serotype through a directional chord. The chord thickness at the arc represents the number of co-colonization events in which that serotype is dominant. The thickness of the arc is related to the relative dominance of the serotype rather than the prevalence. Where more than two serotypes were in co-colonization, these were paired separately with the dominant serotype, e.g., 6A>23F>15BC would be 6A>23F; 6A>15BC. Where the chord terminates in a stroke, e.g., serotype 29 in both panels, this indicates the serotype is dominant in zero cases of co-colonization. The flow of dominance is also represented by a color gradient, where the more often dominant serotype determines the color of the chord. If a chord color is different from the color of the terminating arc, this represents the serotype at the base color of the chord being mostly dominant. An interactive version of each chord diagram is available at https://public.flourish.studio/visualisation/12162597/ and https://public.flourish.studio/visualisation/12182182/.

When detected together with other pneumococcal serotypes in period-1, VT 6A, 19F, 23F, 18C, 19A, and 14 were more frequently the dominant colonizing serotype (Figure 3a, Supplementary Table 6). This is demonstrated by the arc size for the respective serotypes in Figure 3, which is linked to the number of times they are the dominant co-colonizing serotype. Notably, serotypes 19F and 6A were detected as the dominant serotype in 92.0% (69/75) and 92.5% (74/80), respectively, of all occasions they were found co-colonizing with any other serotype in period-1. In period-2, VT 6A, 14, and 19F persisted as the dominant colonizers when carried concurrently with other serotypes, with 19F being the dominant colonizer in 93.5% (43/46) of cases where it was detected in period-2, shown also by the similar arc size in both periods (Figure 3b; Supplementary Table 6). Serotype 6C increased in dominance relative to other co-colonizing serotypes in period-2 (Figure 3b), despite the cross-protection against serotype 6C afforded by the inclusion of 6A in PCV13. In period-2, NVT 8, 9LN, 10A, 10CF, 13, 15AF, 23A, 23B, 35B, 35F, 43, and 47A increased in dominance in co-colonization compared with period-1 and the arc size for these serotypes becomes larger in Figure 3b, representing replacement in co-colonization.

### Colonization prevalence by other bacteria

The only difference in colonization by investigated bacteria in period-2 compared with period-1 was the higher prevalence of *N. Lactamica* (8.9%; 51/571 vs 6.8%; 77/1135; aOR: 2.39; 95% CI: 1.29-4.43); Supplementary Figure 5, Supplementary Table 4. Also, there were no differences in the GMD of non-pneumococcal bacterial colonizers between period-2 and period-1 (Supplementary Figure 7, Supplementary Table 5).

### Co-colonization of other bacterial pathogens

**Figure 4:**
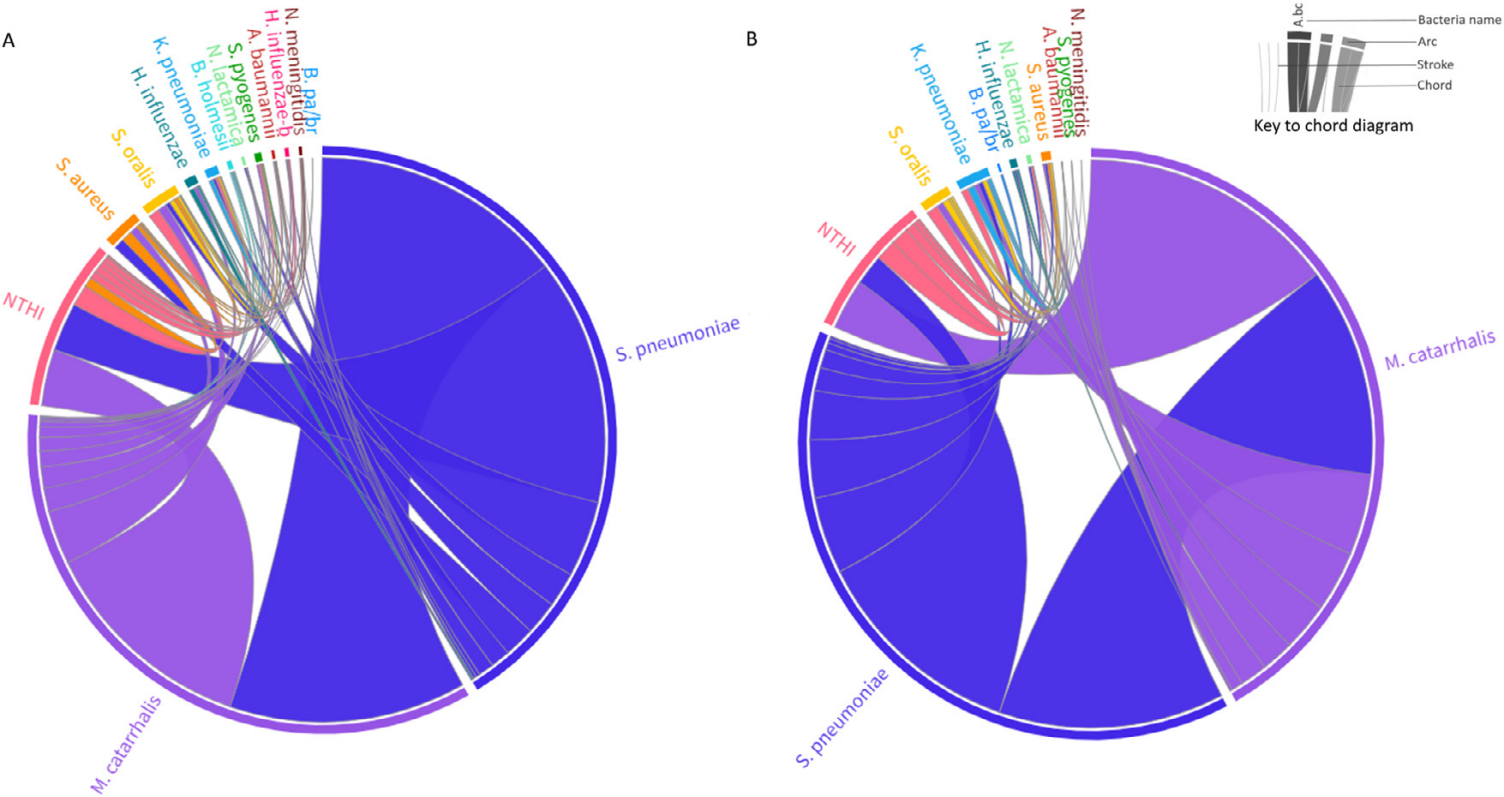
Chord diagram of bacterial co-colonizing events in children 0-60 months of age in Soweto, period-1 and period-2. Panel A includes period-1 (2010), and Panel B includes period-2 (2018). The right inset describes features of the diagram. The outer circle contains arcs that represent each bacterial species. Each arc is paired with a co-colonizing bacterium through a directional chord. The chord thickness at the arc represents the number of co-colonization events in which that bacterium is dominant. The thickness of the arc is related to the relative dominance of the bacterium rather than the prevalence. Where more than two bacteria were in co-colonization, these were paired separately with the dominant bacterium, e.g., Spn>Mca>Hin would be Spn>Mca; Spn>Hin. Where the chord terminates in a stroke, this indicates the bacterium is dominant in zero cases of co-colonization. The flow of dominance is also represented by a color gradient, where the more often dominant bacterium determines the color of the chord. If a chord color is different from the color of the terminating arc, this represents the bacterium at the base color of the chord being mostly dominant. An interactive version of each chord diagram is available at https://public.flourish.studio/visualisation/12186193/ and https://public.flourish.studio/visualisation/12583513/.

A lower percentage of children had more than one bacterial species identified in period-2 (78.1%; 446/571) compared with in period-1 (83.7%; 950/1135; OR: 0.69; 95% CI: 0.5-0.9). In both study periods, *M. catarrhalis* and *S. pneumoniae* were more frequently detected as dominant colonizing bacterium than sub-dominant in co-colonization events, as shown by the large arc size in Figure 4. In contrast, *A. baumannii, H. influenzae, K. pneumoniae, N. lactamica, N. meningitidis, S. aureus, S. oralis,* and *S. pyogenes* were mostly detected as sub-dominant colonizers (Supplementary Table 7) when identified with another bacterial species.

## Discussion

Our study reports a modest decrease in VT nasopharyngeal colonization (54.5%) in children <5 years of age, almost nine years since PCV was first introduced into the South African immunization program, despite 93-100% of the sampled children having been fully vaccinated for their age. When we excluded serotypes 19F and three from the analysis, there was a 73.6% lower prevalence of colonization by the remaining VT in period-2 relative to period-1, with the main circulating serotypes being 5 (2.3%), 6A, 6B, and 19A (all 1.6%).

Despite the reduction in VT, the high carriage prevalence of serotype 19F (8.1%) is a concern, as it accounted for 43.5% of residual VT colonization (18.6%). Furthermore, 19F was more likely to be identified as the highest-density serotype (>80% of the time) than sub-dominant in co-colonized children in both study periods. This is concerning as higher carriage density has been linked to increased transmission and progression to disease [17,18]. Also, 19F is typically carried for longer (212 days) compared with other serotypes (median 60 days) [19], increasing potential for 19F transmission.

The high residual carriage of 19F is corroborated by other colonization studies (1.4-4.5%) in urban settings in sub-Saharan Africa [5,20] and warrants further surveillance and genomic investigation, especially as 19F continues to be associated with ongoing VT IPD in South Africa [21]. Although PCVs reportedly prevent the acquisition of serotype 19F [22], the conjugation method used for PCV13 (reductive amination) may induce less productive opsonophagocytic antibodies against 19F [23]. Coupled with a higher force of infection from a high baseline prevalence, PCVs may not fully interrupt the transmission of 19F in our setting. Similarly, PCV13 had limited effectiveness against serotype 3 IPD [24]. Higher antibody concentrations have been correlated with protection against IPD for serotypes 3 (2.83 μg/ml) and 19F (1.17 μg/ml) than the putative threshold of 0.35 μg/ml when aggregating across all serotypes [24]. Generally, a higher antibody concentration is required to protect against mucosal disease and carriage compared with protection against IPD [25].

Consistently in sub-Saharan Africa, persistent VT colonization after the routine use of PCVs has been documented in urban settings similar to ours, including 9.6% (Congo, 2 years post-PCV) [20]; 11.4% (Gambia, 7 years post-PCV) [5] and 17.5% (Malawi, 4-7 years post-PCV) [4] for PCV13-VT and 6% (Kenya, 5 years post-PCV) for PCV10-VT [6], compared with large reductions in VT (to <3%) typically observed in higher income settings [26]. It is, however, difficult to directly compare these estimates to ours, due to the disparate detection and serotyping schemes used to assess VT and NVT pneumococcal colonization in each study. All four studies were culture-based or utilized an initial culture step. Typically, culture-based typing is not sensitive in the detection of co-colonizing serotypes [12]. Here, we used a sensitive qPCR that could detect 92 serotypes with a limit of detection of 10-100GE/ml, maximizing the ability to detect and serotype pneumococcus, including in co-colonization, and at low density.

In our setting, enhancing protection against the acquisition of 19F, which could translate to further reductions in 19F IPD, is warranted. Of interest, we recently reported that a one-dose primary series of PCV13 and booster dose (14 and 40 weeks of age) yielded higher anti-19F IgG compared with the two-dose and booster schedule currently used in South Africa (6, 14, and 40 weeks of age). Furthermore, the 1+1 schedule was associated with 2.89-fold lower prevalence of 19F colonization at 15 months of age compared with the 2+1 schedule, suggesting that transitioning to a 1+1 schedule could assist in reducing the residual prevalence of 19F colonization in settings such as South Africa [27].

The proportion of colonized children that carried more than one serotype was similar in both periods, ranging from 37.9-40.4%. We detected increases in the frequency of NVT (6C, 8, 9LN, 10A, 10CF, 13, 15AF, 23A, 23B, 35B, 35F, 43, and 47A) being the dominant serotype in co-colonization episodes in period-2, likely due to unmasking when dominant VT was reduced in prevalence. Assessing co-colonization is important in surveillance, as transformation, the ability to acquire genetic elements from other serotypes or bacteria, requires physical proximity afforded by co-colonization [28]. This has implications for antimicrobial resistance genes and capsule switching, the latter to evade serotype-specific vaccine-derived immunity. Indeed, in The Gambia, Multi-Locus-Sequence-Typing, revealed higher proportions of children under-2-years-old carrying multiple serotypes, and genetic transformation (34%) compared with those in higher income settings (2-5%), which the authors attribute to higher carriage prevalence [29]. It will therefore be important to further interrogate episodes of co-colonization for antimicrobial resistance genes.

Limitations of our study, include that our study was not powered to detect changes in less-prevalent individual serotypes, temporally linked with vaccination. As a result, trends in the detection of individual serotypes and density were exploratory. Different swab consumables were used in period-1 and period-2. Nevertheless, this should not have impacted our estimation of bacterial colonization prevalence, as the recovery of *S. pneumoniae* from FLOQSwabs (used in period-2) is comparable to that of Dacron swabs (period-1) [30]. In addition, good diagnostic sensitivity (average 89.1%) has been demonstrated for the qPCR method used here, compared with the culture-based Quellung method, previously used to serotype pneumococcus in period-1 [12]. Still, FLOQSwabs have been shown to yield a higher density [30] and thus comparisons of density (GMD) of colonizing bacteria between period-1 (Dacron swabs) and period-2 (FLOQSwabs) should be interpreted with caution. Furthermore, the previous storage time (10 years) and freeze-thaw cycles for the period-1 samples may have impacted the yield of the NP swabs in our study, meaning we may have underestimated the prevalence or density of some lower-density colonizers. In a comparison between the qPCR method used here and the Quellung method performed soon after sample collection, Quellung detected an additional 9.3% of serotypes [12]. Regardless, when compared with Quellung, qPCR detected almost 40% more serotypes, around 72-90% of which were co-colonizing serotypes [12]. This translates to an overall higher sensitivity to detect co-colonizing and lower-density serotypes, minimizing the effects of storage or freeze-thaw cycles on our conclusions. Although we used the presence of multiple pan-pneumococcal genes to confirm pneumococcus, in the case of co-colonization, some non-pneumococcal species with homologous genetic regions to the targeted pneumococcal capsular regions, may be typed as pneumococcal serotypes, overestimating their prevalence.

This study is important in our setting as South Africa considers the transition to a reduced PCV dosing schedule. Here, we find that in children targeted for routine vaccination (<60 months old) have a reduced risk of disease from VT compared with the baseline period (2010, period-1) and with vaccination coverage above 80% and the possibility that a 1+1 schedule compared with 2+1 schedule could reduce 19F colonization, transitioning to the reduced dosing schedule should be considered.

## Declarations of competing interest

The authors have no competing interests to declare.

## Appendix Supplementary materials

Image, application 1

Image, application 2

Image, application 3

Image, application 4

Image, application 5

Image, application 6

Image, application 7

Image, application 8

Image, application 9

Image, application 10

Image, application 11

Image, application 12

Image, application 13

Image, application 14

Image, application 15
