# Supplementary material for: Streptococcus pneumoniae and other bacterial nasopharyngeal colonization seven years post-introduction of 13-valent pneumococcal conjugate vaccine in South African children
Source: Int J Infect Dis. 2023 Sep;134:45–52. doi: 10.1016/j.ijid.2023.05.016 (PMC10404162; doi:10.1016/j.ijid.2023.05.016)
Supplement: Supplementary file 1 [file mmc1.docx]

**Supplementary Figure 1:** Administrative and Official Pneumococcal vaccination coverage in South Africa reported annually through the World Health Organization (WHO)/ The United Nations Children's Fund (UNICEF) Joint Reporting Form on Immunization (JRF) and the WHO and UNICEF Joint Estimates of National Immunization Coverage (WUENIC) ^(1, 2)^.
*WUENIC estimates include the percentage of surviving infants who received the 3rd dose of pneumococcal conjugate vaccine or infants that received at least 2 doses of PCV prior to their first birthday. Official estimates are usually based on data from the administrative method. Administrative data is calculated based on the number of doses recorded as administered as part of the national immunization schedule. The national Department of Health may provide official estimates from other sources. Official and Administrative data been combined here. For the Official/Administrative data, PCV 1; PCV 2 and PCV 3 is the percentage in the target population who have received one; two and three doses of PCV respectively in each year.*

1. Kowalski R. BA. WUENIC – A Case Study in Rule-Based Knowledge Representation and Reasoning. In: Okumura M., Bekki D., Satoh K. (eds) New Frontiers in Artificial Intelligence. JSAI-isAI 2011. . Lecture Notes in Computer Science, Springer, Berlin, Heidelberg. 2012;vol 7258.

2. Organization WH. WHO Immunization Data Dashboard: Pneumococcal vaccination coverage. <https://immunizationdatawhoint/pages/coverage/PCVhtml>. 2021;Accessed, June 2021.
