# Supplementary material for: Streptococcus pneumoniae and other bacterial nasopharyngeal colonization seven years post-introduction of 13-valent pneumococcal conjugate vaccine in South African children
Source: Int J Infect Dis. 2023 Sep;134:45–52. doi: 10.1016/j.ijid.2023.05.016 (PMC10404162; doi:10.1016/j.ijid.2023.05.016)
Supplement: Supplementary file 2 [file mmc2.docx]

**
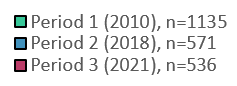
**
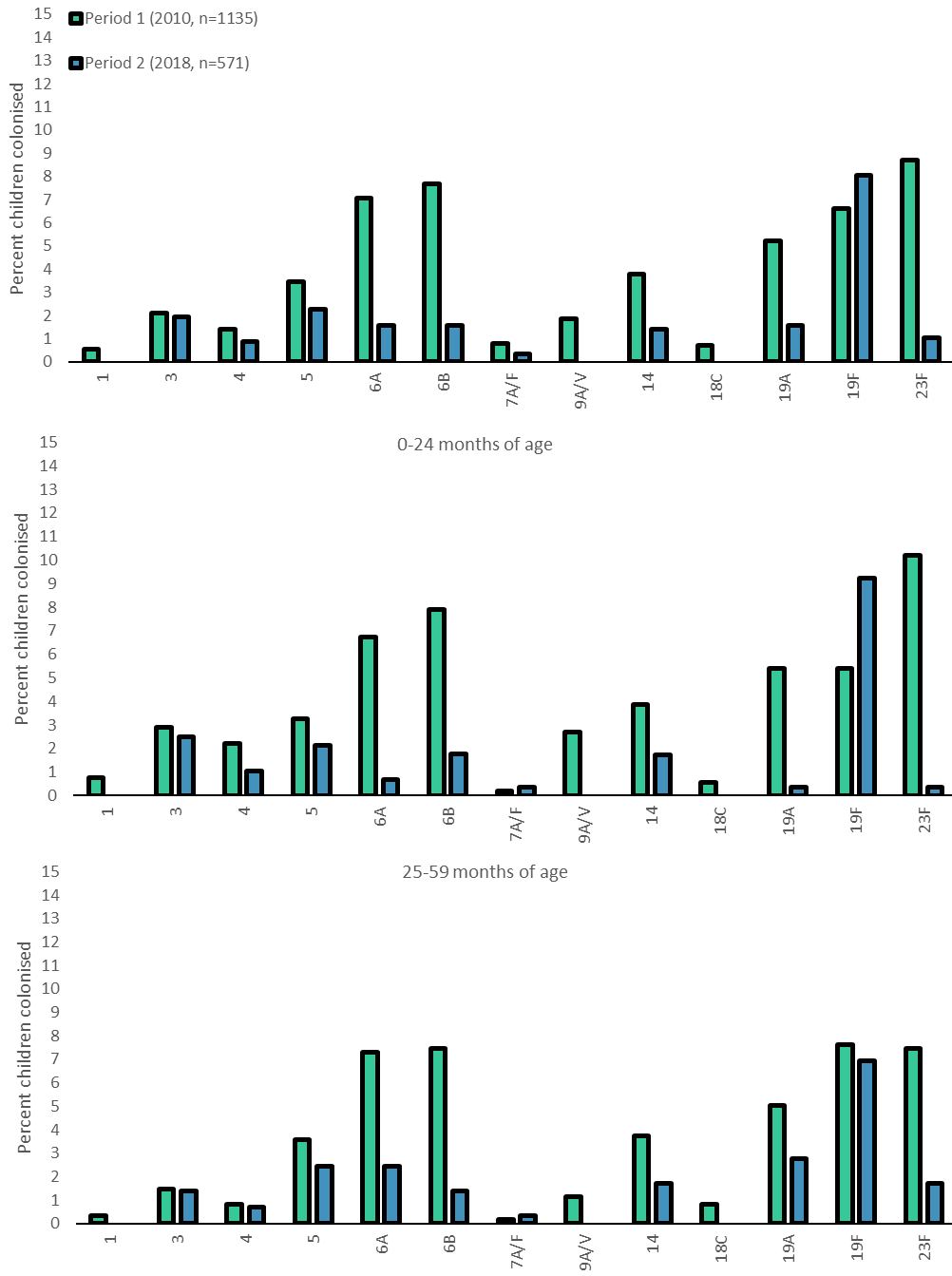
*
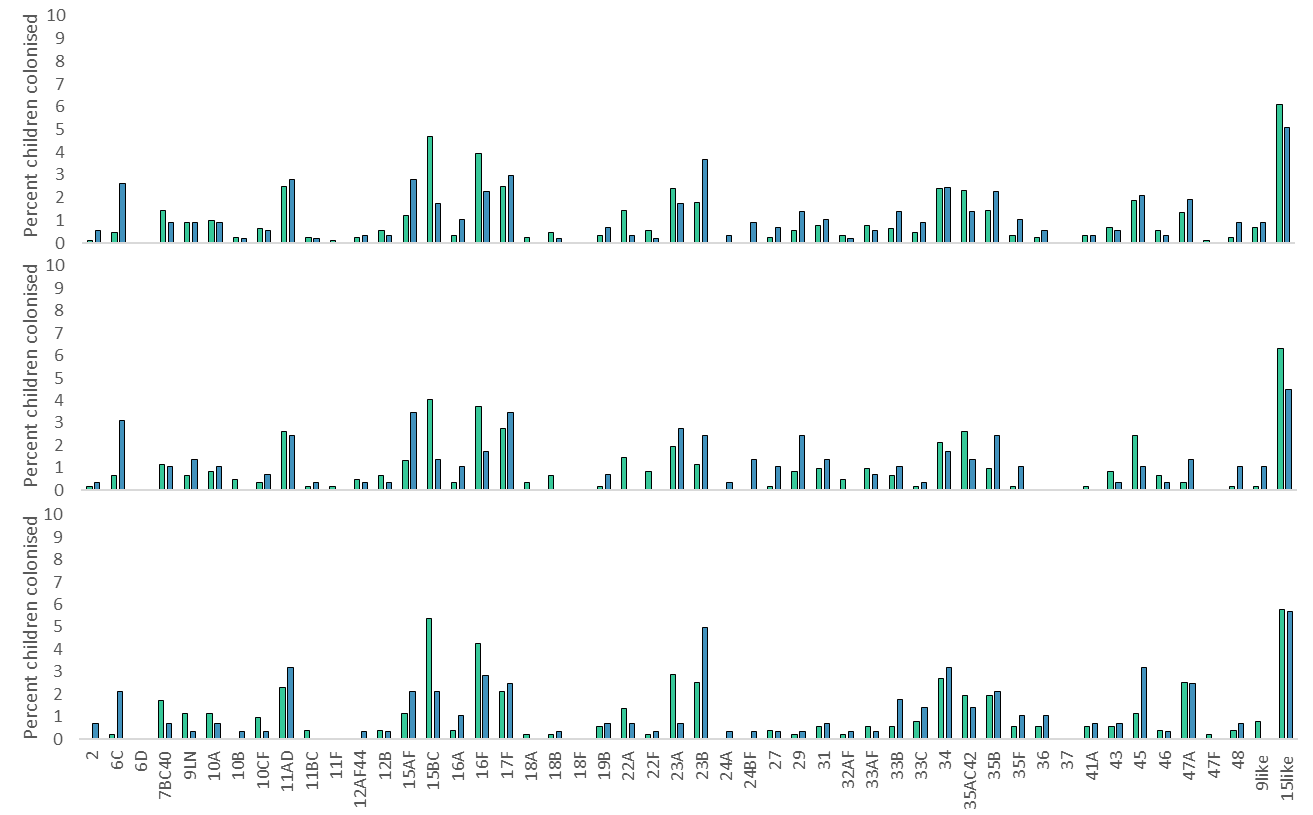
*

A

B

C

**Supplementary Figure 2:** Prevalence of non-vaccine serotypes in children 0-60 months-of-age.
*Panel A includes all children 0-60 months-of-age, Panel B includes children 0-24 months-of-age and Panel C includes children 25-60 months-of-age;* *p-values <0.01 were considered significant. Adjusted Odds Ratios and p-values calculated using logistic regression in Supp table 2.*
