# Supplementary material for: Streptococcus pneumoniae and other bacterial nasopharyngeal colonization seven years post-introduction of 13-valent pneumococcal conjugate vaccine in South African children
Source: Int J Infect Dis. 2023 Sep;134:45–52. doi: 10.1016/j.ijid.2023.05.016 (PMC10404162; doi:10.1016/j.ijid.2023.05.016)
Supplement: Supplementary file 3 [file mmc3.docx]

**
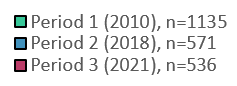

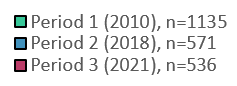
**
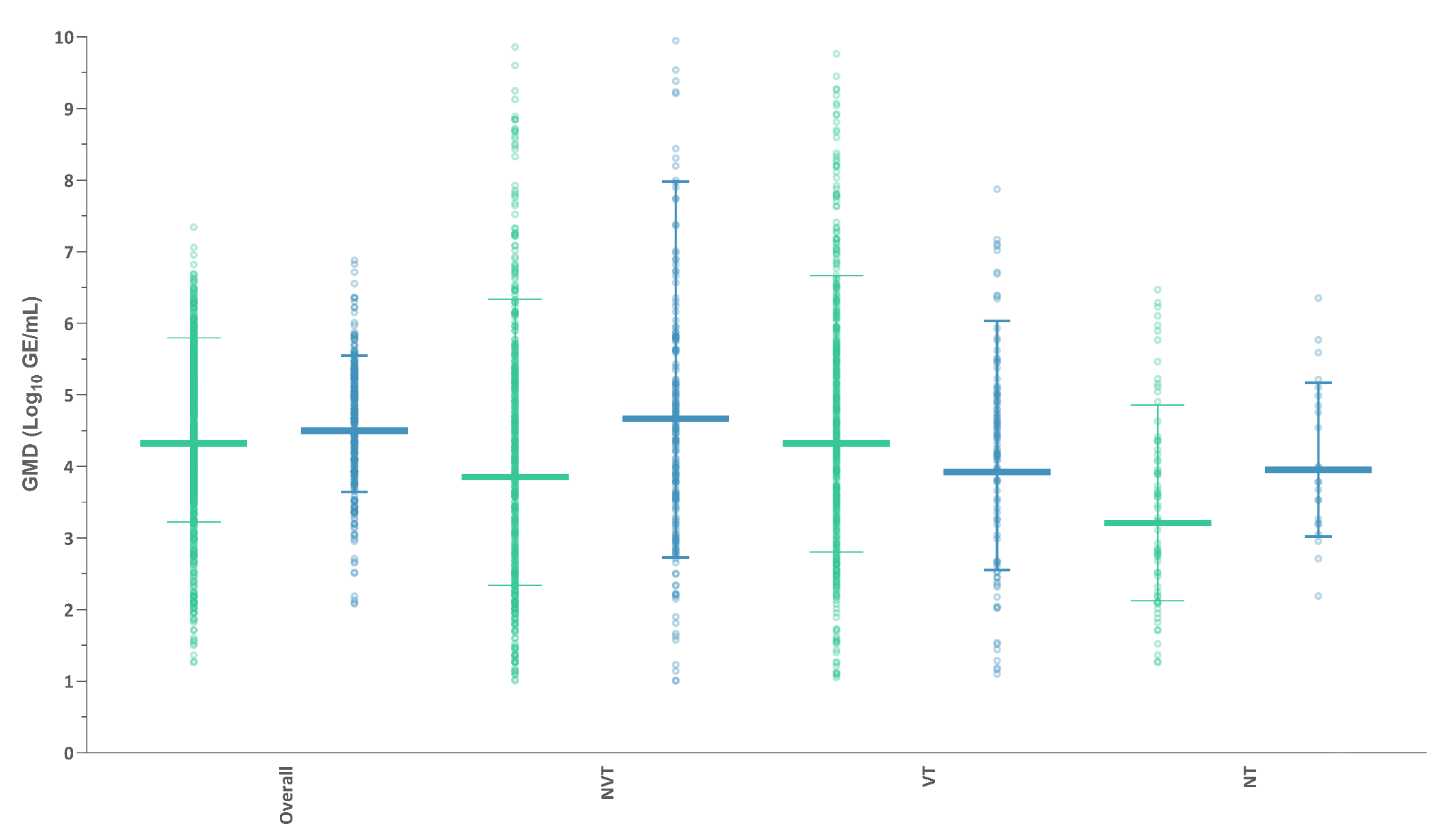


**p<0.001**

**p=0.010**

**Supplementary Figure 3**: Geometric mean density (GMD, log_10_ Genomic Equivalents per mL [GE/mL]) of *Streptococcus pneumoniae* in children 0-60 months of age.
*Overall includes all pneumococcus; VT include PCV13 vaccine serotypes serotypes/serogroups 1, 3, 4, 5,6A, 6B, 7A/F, 9A/V, 14, 18C, 19A, 19F and 23F; NVT, all serotypes/serogroups not included in PCV13. NTSP, non-typeable S. pneumoniae. Only significant p-values shown, p-values <0.01 were considered significant. All other p-values presented in supp table 3.*
