# Supplementary material for: Streptococcus pneumoniae and other bacterial nasopharyngeal colonization seven years post-introduction of 13-valent pneumococcal conjugate vaccine in South African children
Source: Int J Infect Dis. 2023 Sep;134:45–52. doi: 10.1016/j.ijid.2023.05.016 (PMC10404162; doi:10.1016/j.ijid.2023.05.016)
Supplement: Supplementary file 4 [file mmc4.docx]

**
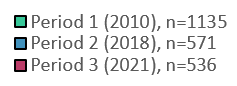
**
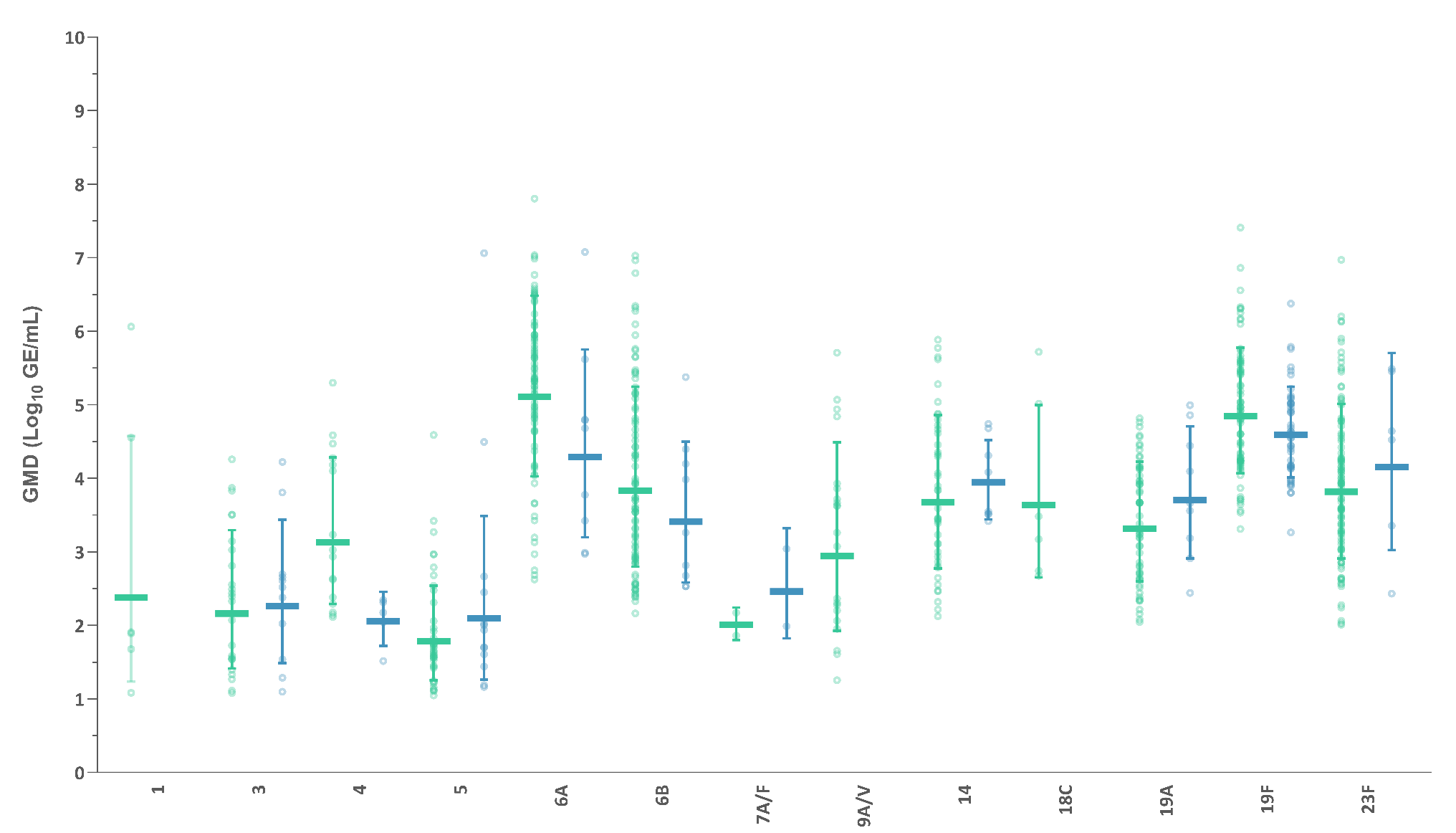


**p=0.005**

**p=0.003**

**p=0.002**

**p<0.001**

**Supplementary Figure 4**: Geometric mean density (GMD log_10_ Genomic Equivalents per mL [GE/mL]) of colonising PCV13 vaccine serotypes in children 0-60 months-of-age.
*Only significant p-values shown, p-values <0.01 were considered significant. All other p-values presented in supp table 3.*
