# Supplementary material for: Streptococcus pneumoniae and other bacterial nasopharyngeal colonization seven years post-introduction of 13-valent pneumococcal conjugate vaccine in South African children
Source: Int J Infect Dis. 2023 Sep;134:45–52. doi: 10.1016/j.ijid.2023.05.016 (PMC10404162; doi:10.1016/j.ijid.2023.05.016)
Supplement: Supplementary file 5 [file mmc5.docx]

**
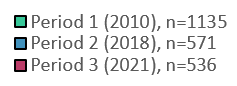


**

p=0.008

**

**

p=0.014

**Supplementary Figure 5:** Geometric mean density (GMD log_10_ Genomic Equivalents per mL [GE/mL]) of non-PCV13 serotypes (NVT) in children 0-60 months of age. *Only significant values (<0.01) presented. All* *p-values in Supp table 3.*
