# Supplementary material for: Streptococcus pneumoniae and other bacterial nasopharyngeal colonization seven years post-introduction of 13-valent pneumococcal conjugate vaccine in South African children
Source: Int J Infect Dis. 2023 Sep;134:45–52. doi: 10.1016/j.ijid.2023.05.016 (PMC10404162; doi:10.1016/j.ijid.2023.05.016)
Supplement: Supplementary file 7 [file mmc7.docx]

**
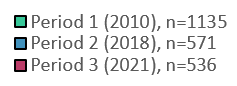
**
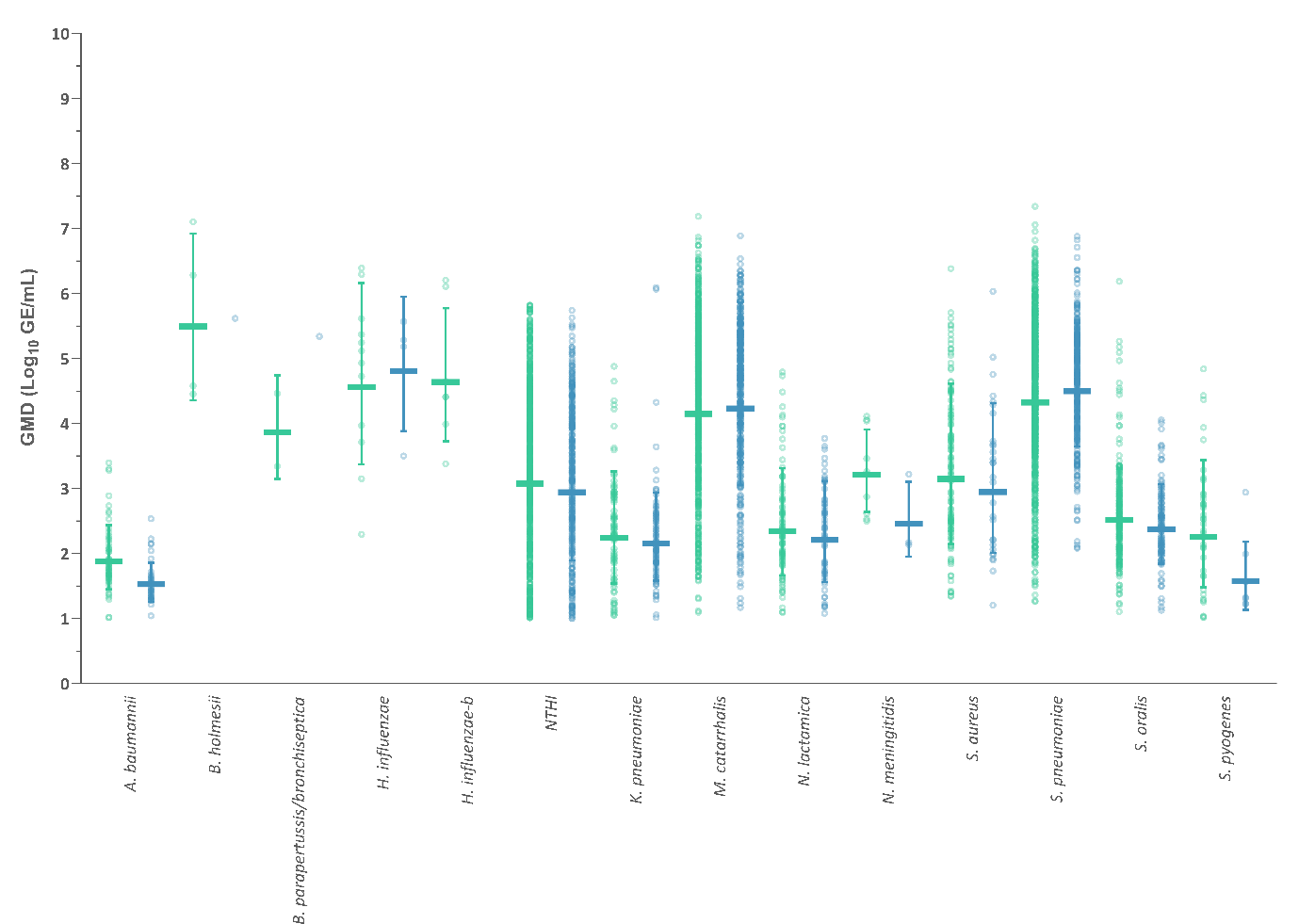


**Supplementary Figure 7:** Geometric mean density (GMD log_10_ Genomic Equivalents per mL [GE/mL]) of bacterial colonisers in children 0-60 months of age across three study periods.
*Only significant p-values shown, p-values <0.01 were considered significant. All other p-values presented in supp table 5.*
