# Supplementary material for: Streptococcus pneumoniae and other bacterial nasopharyngeal colonization seven years post-introduction of 13-valent pneumococcal conjugate vaccine in South African children
Source: Int J Infect Dis. 2023 Sep;134:45–52. doi: 10.1016/j.ijid.2023.05.016 (PMC10404162; doi:10.1016/j.ijid.2023.05.016)
Supplement: Supplementary file 8 [file mmc8.docx]

**Supplementary Table 1:** Pneumococcal conjugate vaccine coverage in Soweto, Gauteng in Period-2 (2018)

| **Age (weeks)** | **1 dose** | **2 doses** | **3 doses** |
| --- | --- | --- | --- |
| 6-14 % (n/N) | 100 (24/24) | - | - |
| 14-40 % (n/N) | 98.6 (68/69) | 100 (69/69) | - |
| >40 % (n/N) | 99.2 (475/479) | 98.3 (471/479) | 93.3 (477/479) |
