# Supplementary material for: Streptococcus pneumoniae and other bacterial nasopharyngeal colonization seven years post-introduction of 13-valent pneumococcal conjugate vaccine in South African children
Source: Int J Infect Dis. 2023 Sep;134:45–52. doi: 10.1016/j.ijid.2023.05.016 (PMC10404162; doi:10.1016/j.ijid.2023.05.016)
Supplement: Supplementary file 9 [file mmc9.docx]

**Supplementary Table 2**: Prevalence of *Streptococcus pneumoniae* colonisation in Period-1 (2010) and Period-2 (2018) in Sowetan children 0-60 months-of-age.

|  | **Age Group**  **Months** | **Period-2  % (n); N=571** | **Period-1  % (n); N=1135** | **OR (95% CI); p-value** | **aOR (95% CI); p-value** |
| --- | --- | --- | --- | --- | --- |
| **Overall pneumococcus** | 0-60 | 49.4 (282) | 68.1 (773) | 0.46 (0.37-0.56); p<0.001 | 0.66 (0.54-0.88); p=0.004 |
|  | 0-24 | 50.9 (147) | 66.1 (407) | 0.53 (0.4-0.7); p<0.001 | 0.69 (0.47-1); p=0.05 |
|  | 25-60 | 47.9 (135) | 57.2 (366) | 0.38 (0.28-0.52); p<0.001 | 0.58 (0.38-0.9); p=0.015 |
| **PCV13-VT** | 0-60 | 18.6 (106) | 40.9 (465) | 0.33 (0.26-0.42); p<0.001 | 0.41 (0.3-0.56); p<0.001 |
|  | 0-24 | 17.4 (49) | 42.6 (221) | 0.38 (0.27-0.52); p<0.001 | 0.41 (0.27-0.62); p<0.001 |
|  | 25-60 | 17.4 (49) | 42.6 (221) | 0.28 (0.2-0.4); p<0.001 | 0.37 (0.23-0.6); p<0.001 |
| **NVT** | 0-60 | 37.8 (216) | 42.4 (481) | 0.83 (0.67-1.01); p=0.07 | 0.96 (0.72-1.26); p=0.75 |
|  | 0-24 | 38.4 (111) | 40.4 (249) | 0.92 (0.69-1.22); p=0.56 | 1.06 (0.72-1.55); p=0.79 |
|  | 25-60 | 37.2 (105) | 44.7 (232) | 0.73 (0.54-0.98); p=0.04 | 0.87 (0.57-1.32); p=0.53 |
| **NT** | 0-60 | 5.95 (34) | 7.58 (86) | 0.77 (0.51-1.16); p=0.22 | 0.99 (0.57-1.71); p=0.98 |
|  | 0-24 | 7.6 (22) | 7.5 (46) | 1.02 (0.6-1.74); p=0.94 | 1.29 (0.62-2.71); p=0.49 |
|  | 25-60 | 4.3 (12) | 7.7 (40) | 0.53 (0.27-1.03); p=0.06 | 0.69 (0.28-1.68); p=0.41 |
| **1** | 0-60 | 0 (0) | 0.53 (6) | p=0.08 | - |
|  | 0-24 | 0 (0) | 0.32 (2) | p=0.35 | - |
|  | 25-60 | 0 (0) | 0.77 (4) | p=0.15 | - |
| **3** | 0-60 | 1.93 (11) | 2.11 (24) | 0.91 (0.44-1.87); p=0.8 | 2.3 (0.63-8.19); p=0.21 |
|  | 0-24 | 1.38 (4) | 1.46 (9) | 0.94 (0.29-3.1); p=0.93 | 2.49 (0.2-6.24) p=0.43 |
|  | 25-60 | 2.48 (7) | 2.89 (15) | 0.86 (0.34-2.12); p=0.74 | 2.09 (0.42-10.38) p=0.37 |
| **4** | 0-60 | 0.88 (5) | 1.41 (16) | 0.62 (0.23-1.7); p=0.35 | 0.6 (0.19-2.16); p=0.47 |
|  | 0-24 | 0.69 (2) | 0.81 (5) | 0.85 (0.16-4.42); p=0.85 | 1.29 (0.12-14.29) p=0.84 |
|  | 25-60 | 1.06 (3) | 2.21 (11) | 0.5 (0.14-1.8); p=0.29 | 0.53 (0.11-2.53) p=0.43 |
| **5** | 0-60 | 2.28 (13) | 3.44 (39) | 0.65 (0.35-1.24); p=0.19 | 0.5 (0.23-1.06); p=0.07 |
|  | 0-24 | 2.42 (7) | 3.57 (22) | 0.67 (0.28-1.59); p=0.36 | 0.51 (0.18-1.46) p=0.21 |
|  | 25-60 | 2.13 (6) | 3.28 (17) | 0.64 (0.25-1.65); p=0.36 | 0.47 (0.14-1.57) p=0.22 |
| **6A** | 0-60 | 1.58 (9) | 7.05 (80) | 0.21 (0.11-0.42); p<0.001 | 0.2 (0.1-0.49); p<0.001 |
|  | 0-24 | 2.42 (7) | 7.31 (45) | 0.31 (0.14-0.71); p=0.005 | 0.37 (0.14-0.99) p=0.048 |
|  | 25-60 | 0.71 (2) | 6.74 (35) | 0.1 (0.02-0.41); p=0.002 | 0.09 (0.01-0.34) p=0.001 |
| **6B** | 0-60 | 1.58 (9) | 7.67 (87) | 0.19 (0.1-0.39); p<0.001 | 0.3 (0.11-0.56); p<0.001 |
|  | 0-24 | 1.38 (4) | 7.47 (46) | 0.17 (0.06-0.48); p=0.001 | 0.26 (0.08-0.87) p=0.029 |
|  | 25-60 | 1.77 (5) | 7.90 (41) | 0.21 (0.08-0.54); p=0.001 | 0.22 (0.07-0.67) p=0.008 |
| **7A/F** | 0-60 | 0.35 (2) | 0.18 (2) | 1.99 (0.28-14.18); p=0.49 | 1.1 (0.1-12.6); p=0.93 |
|  | 0-24 | 0.35 (1) | 0.16 (1) | 2.13 (0.13-34.26); p=0.59 | 0.64 (0.04-10.51) p=0.75 |
|  | 25-60 | 0.35 (1) | 0.19 (1) | 1.84 (0.11-29.58); p=0.66 | 9.2 (0.03-272.48) p=0.46 |
| **9A/V** | 0-60 | 0 (0) | 1.85 (21) | p=0.001 | - |
|  | 0-24 | 0 (0) | 1.14 (1) | p=0.07 | - |
|  | 25-60 | 0 (0) | 2.7 (14) | p=0.005 | - |
| **14** | 0-60 | 1.4 (8) | 3.79 (43) | 0.36 (0.17-0.77); p=0.009 | 0.4 (0.15-0.92); p=0.03 |
|  | 0-24 | 1.73 (5) | 3.73 (23) | 0.45 (0.17-1.2); p=0.11 | 0.44 (0.14-1.43) p=0.17 |
|  | 25-60 | 1.73 (5) | 3.85 (20) | 0.27 (0.08-0.91); p=0.04 | 0.32 (0.07-1.39) p=0.13 |
| **18C** | 0-60 | 0 (0) | 0.7 (8) | p=0.004 | - |
|  | 0-24 | 0 (0) | 0.81 (5) | p=0.13 | - |
|  | 25-60 | 0 (0) | 0.58 (3) | p=0.2 | - |
| **19A** | 0-60 | 1.58 (9) | 5.2 (59) | 0.29 (0.14-0.59); p=0.001 | 0.3 (0.12-0.61); p<0.001 |
|  | 0-24 | 2.77 (8) | 5.03 (31) | 0.54 (0.24-1.19); p=0.12 | 0.35 (0.14-0.87) p=0.02 |
|  | 25-60 | 0.35 (1) | 5.39 (28) | 0.06 (0.008-0.46); p=0.007 | 0.11 (0.01-0.91) p=0.04 |
| **19F** | 0-60 | 8.06 (46) | 6.61 (75) | 1.23 (0.85-1.81); p=0.27 | 2 (1.09-3.56); p=0.03 |
|  | 0-24 | 6.92 (20) | 7.63 (47) | 0.9 (0.52-1.55); p=0.7 | 0.23 (0.5-72.8) p=0.56 |
|  | 25-60 | 9.22 (26) | 5.39 (28) | 1.78 (1.0 -3.1); p=0.04 | 2.45 (0.93-6.41) p=0.07 |
| **23F** | 0-60 | 1.05 (6) | 8.72 (99) | 0.11 (0.05-0.25); p<0.001 | 0.2 (0.07-0.41); p<0.001 |
|  | 0-24 | 1.73 (5) | 7.47 (46) | 0.22 (0.09-0.56); p=0.001 | 0.27 (0.09-0.78) p=0.02 |
|  | 25-60 | 0.35 (1) | 10.21 (53) | 0.03 (0.004-0.23); p=0.001 | 0.05 (0.01-0.41) p=0.005 |
| **2** | 0-60 | 0.53 (3) | 0.09 (1) | 5.99 (0.62-57.71); p=0.12 | - |
|  | 0-24 | 0.35 (1) | 0.16 (1) | 2.14 (0.13-34.26); p=0.59 | 1.16 (0.79-1.72); p=0.45 |
|  | 25-60 | 0.71 (2) | 0 (0) | - | - |
| **6C** | 0-60 | 2.63 (15) | 0.44 (5) | 6.1 (2.2-16.86); p<0.001 | 4.57 (1.05-19.93); p = 0.04 |
|  | 0-24 | 3.11 (9) | 0.65 (4) | 4.92 (1.5-16.1); p=0.008 | 2.76 (0.58-13.01); p=0.2 |
|  | 25-60 | 2.13 (6) | 0.19 (1) | 11.26 (1.35-94.01); p=0.025 | 47.97 (0.77-2996.83); p=0.07 |
| **7B/7C/40** | 0-60 | 0.88 (5) | 1.41 (16) | 0.62 (0.23-1.7); p=0.35 | 1.58 (0.31-8); p = 0.58 |
|  | 0-24 | 1.04 (3) | 1.14 (7) | 0.91 (0.23-3.55); p=0.9 | 40.27 (0.04-37553.68); p=0.29 |
|  | 25-60 | 0.71 (2) | 1.73 (9) | 0.4 (0.09-1.89); p=0.25 | 0.61 (0.08-4.68); p=0.63 |
| **9L/9N** | 0-60 | 0.88 (5) | 0.88 (10) | 0.99 (0.34-2.92); p=0.99 | 0.86 (0.22-3.44); p = 0.83 |
|  | 0-24 | 1.38 (4) | 0.65 (4) | 2.15 (0.53-8.65); p=0.28 | 1.96 (0.21-18.23); p=0.56 |
|  | 25-60 | 0.35 (1) | 1.16 (6) | 0.3 (0.04-2.54); p=0.27 | 0.37 (0.03-3.91); p=0.41 |
| **10A** | 0-60 | 0.88 (5) | 0.97 (11) | 0.9 (0.31-2.61); p=0.85 | 0.9 (0.23-3.62); p = 0.89 |
|  | 0-24 | 1.04 (3) | 0.81 (5) | 1.28 (0.3-5.4); p=0.74 | 1.45 (0.14-14.77); p=0.75 |
|  | 25-60 | 0.71 (2) | 1.16 (6) | 0.61 (0.12-3.05); p=0.55 | 0.74 (0.11-5.19); p=0.76 |
| **10B** | 0-60 | 0.18 (1) | 0.26 (3) | 0.66 (0.07-6.38); p=0.72 | 0.2 (0.02-2.47); p = 0.21 |
|  | 0-24 | 0 (0) | 0.49 (3) | - | - |
|  | 25-60 | 0.35 (1) | 0 (0) | - | - |
| **10CF** | 0-60 | 0.53 (3) | 0.62 (7) | 0.85 (0.22-3.3); p=0.82 | 0.62 (0.12-3.29); p = 0.57 |
|  | 0-24 | 0.69 (2) | 0.32 (2) | 2.14 (0.3-15.26); p=0.45 | - |
|  | 25-60 | 0.35 (1) | 0.96 (5) | 0.37 (0.04-3.15); p=0.36 | 0.11 (0.01-1.49); p=0.1 |
| **11AD** | 0-60 | 2.80 (16) | 2.47 (28) | 1.14 (0.61-2.12); p=0.68 | 0.95 (0.43-2.08); p = 0.9 |
|  | 0-24 | 2.42 (7) | 2.6 (16) | 0.93 (0.38-2.29); p=0.88 | 0.62 (0.21-1.85); p=0.39 |
|  | 25-60 | 3.19 (9) | 2.31 (12) | 1.39 (0.58-3.35); p=0.46 | 1.87 (0.57-6.15); p=0.3 |
| **11BC** | 0-60 | 0.18 (1) | 0.26 (3) | 0.66 (0.07-6.38); p=0.72 | - |
|  | 0-24 | 0.35 (1) | 0.16 (1) | 2.14 (0.13-34.26); p=0.59 | - |
|  | 25-60 | 0.00 (0) | 0.39 (2) | - | - |
| **11F** | 0-60 | 0 (0) | 0.09 (1) | p=0.48 | - |
|  | 0-24 | 0 (0) | 0.16 (1) | - | - |
|  | 25-60 | 0 (0) | 0 (0) | - | - |
| **12A/12F/44** | 0-60 | 0.35 (2) | 0.26 (3) | 1.33 (0.22-7.96); p=0.76 | 0.94 (0.08-10.88); p = 0.96 |
|  | 0-24 | 0.35 (1) | 0.49 (3) | 0.71 (0.07-6.85); p=0.77 | 0.27 (0.01-5.2); p=0.38 |
|  | 25-60 | 0.35 (1) | 0 (0) | - | - |
| **12B** | 0-60 | 0.35 (2) | 0.53 (6) | 0.66 (0.13-3.29); p=0.61 | 0.56 (0.07-4.27); p = 0.58 |
|  | 0-24 | 0.35 (1) | 0.65 (4) | 0.53 (0.06-4.77); p=0.57 | 0.39 (0.02-6.82); p=0.52 |
|  | 25-60 | 0.35 (1) | 0.39 (2) | 0.92 (0.08-10.19); p=0.95 | 1.25 (0.06-24.85); p=0.89 |
| **15A/15F** | 0-60 | 2.80 (16) | 1.23 (14) | 2.31 (1.12-4.76); p=0.02 | 5.16 (1.27-20.88); p=0.02 |
|  | 0-24 | 3.46 (10) | 1.30 (8) | 2.72 (1.06-6.98); p=0.037 | 6.07 (0.84-44.11); p=0.08 |
|  | 25-60 | 2.13 (6) | 1.16 (6) | 1.86 (0.59-5.82); p=0.29 | 5.54 (0.69-44.7); p=0.11 |
| **15B/15C** | 0-60 | 1.75 (10) | 4.67 (53) | 0.36 (0.18-0.72); p=0.004 | 0.37 (0.16-0.83); p=0.02 |
|  | 0-24 | 1.38 (4) | 4.06 (25) | 0.33 (0.11-0.96); p=0.042 | 0.3 (0.09-1.02); p=0.05 |
|  | 25-60 | 2.13 (6) | 5.39 (28) | 0.38 (0.16-0.93); p=0.034 | 0.4 (0.13-1.22); p=0.11 |
| **16A** | 0-60 | 1.05 (6) | 0.35 (4) | 3 (0.84-10.68); p=0.09 | 44.43 (0.28-6987.84); p=0.14 |
|  | 0-24 | 1.04 (3) | 0.32 (2) | 3.22 (0.54-19.38); p=0.2 | 78.53 (0.02-665994.7); p=0.347 |
|  | 25-60 | 1.06 (3) | 0.39 (2) | 2.78 (0.46-16.73); p=0.26 | - |
| **16F** | 0-60 | 2.28 (13) | 3.96 (45) | 0.56 (0.3-1.05); p=0.07 | 0.83 (0.35-1.98); p = 0.67 |
|  | 0-24 | 1.73 (5) | 3.73 (23) | 0.45 (0.17-1.21); p=0.11 | 0.52 (0.16-1.75); p=0.29 |
|  | 25-60 | 2.84 (8) | 4.24 (22) | 0.66 (0.29-1.5); p=0.32 | 1.07 (0.27-4.31); p=0.92 |
| **17F** | 0-60 | 2.98 (17) | 2.47 (28) | 1.21 (0.66-2.24); p=0.54 | 1.17 (0.5-2.75); p = 0.72 |
|  | 0-24 | 3.46 (10) | 2.76 (17) | 1.26 (0.57-2.79); p=0.56 | 1.25 (0.42-3.68); p=0.69 |
|  | 25-60 | 2.48 (7) | 2.12 (11) | 1.18 (0.45-3.07); p=0.74 | 1.27 (0.3-5.38); p=0.74 |
| **18A** | 0-60 | 0.00 (0) | 0.26 (3) | p=0.22 | - |
|  | 0-24 | 0 (0) | 0.32 (2) | - | - |
|  | 25-60 | 0.00 (0) | 0.19 (1) | - | - |
| **18B** | 0-60 | 0.18 (1) | 0.44 (5) | 0.4 (0.05-3.4); p=0.38 | 1.08 (0.06-18.79); p = 0.96 |
|  | 0-24 | 0 (0) | 0.65 (4) | - | - |
|  | 25-60 | 0.35 (1) | 0.19 (1) | 1.84 (0.11-29.58); p=0.67 | 2.14 (0.12-38.14); p=0.6 |
| **19B** | 0-60 | 0.70 (4) | 0.35 (4) | 1.99 (0.50-8.01); p=0.33 | 9.2 (0.34-249.32); p = 0.19 |
|  | 0-24 | 0.69 (2) | 0.16 (1) | 4.29 (0.39-47.46); p=0.24 | - |
|  | 25-60 | 0.71 (2) | 0.58 (3) | 1.23 (0.2-7.4); p=0.82 | - |
| **22A** | 0-60 | 0.35 (2) | 1.41 (16) | 0.25 (0.06-1.07); p=0.06 | 0.22 (0.04-1.15); p = 0.07 |
|  | 0-24 | 0 (0) | 1.46 (9) | - | - |
|  | 25-60 | 0.71 (2) | 1.35 (7) | 0.52 (0.11-2.53); p=0.42 | 0.42 (0.05-3.45); p=0.42 |
| **22F** | 0-60 | 0.18 (1) | 0.53 (6) | 0.33 (0.04-2.75); p=0.31 | 0.31 (0.02-5.33); p = 0.42 |
|  | 0-24 | 0 (0) | 0.81 (5) | - | - |
|  | 25-60 | 0.35 (1) | 0.19 (1) | 1.84 (0.11-29.58); p=0.67 | 0.22 (0.01-4.93); p=0.34 |
| **23A** | 0-60 | 1.75 (10) | 2.38 (27) | 0.73 (0.35-1.52); p=0.4 | 0.6 (0.25-1.43); p = 0.25 |
|  | 0-24 | 2.77 (8) | 1.95 (12) | 1.43 (0.58-3.54); p=0.44 | 0.71 (0.23-2.15); p=0.54 |
|  | 25-60 | 0.71 (2) | 2.89 (15) | 0.24 (0.05-1.06); p=0.06 | 0.28 (0.05-1.54); p=0.14 |
| **23B** | 0-60 | 3.68 (21) | 1.76 (20) | 2.13 (1.14-3.96); p=0.02 | 2.8 (1.05-7.49); p = 0.04 |
|  | 0-24 | 2.42 (7) | 1.14 (7) | 2.16 (0.75-6.22); p=0.15 | 4.43 (0.53-37.35); p=0.17 |
|  | 25-60 | 4.96 (14) | 2.50 (13) | 2.03 (0.94-4.39); p=0.07 | 2.46 (0.77-7.87); p=0.13 |
| **24A** | 0-60 | 0.35 (2) | 0.00 (0) | p=0.05 | - |
|  | 0-24 | 0.35 (1) | 0 (0) | - | - |
|  | 25-60 | 0.35 (1) | 0.00 (0) | - | - |
| **24B/24F** | 0-60 | 0.88 (5) | 0.00 (0) | p=0.02 | - |
|  | 0-24 | 1.38 (4) | 0 (0) | - | - |
|  | 25-60 | 0.35 (1) | 0.00 (0) | - | - |
| **27** | 0-60 | 0.70 (4) | 0.26 (3) | 2.66 (0.59-11.93); p=0.2 | 31.94 (0.4-2525.83); p = 0.12 |
|  | 0-24 | 1.04 (3) | 0.16 (1) | 6.45 (0.67-62.29); p=0.11 | - |
|  | 25-60 | 0.35 (1) | 0.39 (2) | 0.92 (0.08-10.19); p=0.95 | - |
| **29** | 0-60 | 1.40 (8) | 0.53 (6) | 2.67 (0.92-7.74); p=0.07 | 4.58 (0.71-29.58); p = 0.11 |
|  | 0-24 | 2.42 (7) | 0.81 (5) | 3.03 (0.95-9.64); p=0.06 | 4.33 (0.61-360.46); p=0.14 |
|  | 25-60 | 0.35 (1) | 0.19 (1) | 1.84 (0.11-29.58); p=0.67 | - |
| **31** | 0-60 | 1.05 (6) | 0.79 (9) | 1.33 (0.47-3.75); p=0.59 | 0.81 (0.21-3.11); p = 0.76 |
|  | 0-24 | 1.38 (4) | 0.97 (6) | 1.43 (0.4-5.1); p=0.58 | 0.98 (0.17-5.59); p=0.98 |
|  | 25-60 | 0.71 (2) | 0.58 (3) | 1.23 (0.2-7.4); p=0.82 | 0.79 (0.08-7.7); p=0.84 |
| **32A/32F** | 0-60 | 0.18 (1) | 0.35 (4) | 0.5 (0.06-4.45); p=0.53 | 3.87 (0.07-208.35); p = 0.51 |
|  | 0-24 | 0 (0) | 0.49 (3) | - | - |
|  | 25-60 | 0.35 (1) | 0.19 (1) | 1.84 (0.11-29.58); p=0.67 | - |
| **33A/33F** | 0-60 | 0.53 (3) | 0.79 (9) | 0.66 (0.18-2.45); p=0.54 | 1.27 (0.15-10.5); p = 0.83 |
|  | 0-24 | 0.69 (2) | 0.97 (6) | 0.71 (0.14-3.53); p=0.67 | 1.02 (0.1-10.76); p=0.99 |
|  | 25-60 | 0.35 (1) | 0.58 (3) | 0.61 (0.06-5.91); p=0.67 | 4.19 (0.04-474.85); p=0.55 |
| **33B** | 0-60 | 1.40 (8) | 0.62 (7) | 2.29 (0.83-6.35); p=0.11 | 5.32 (0.79-35.28); p = 0.09 |
|  | 0-24 | 1.04 (3) | 0.65 (4) | 1.6 (0.36-7.22); p=0.54 | 8.36 (0.22-311.17); p=0.25 |
|  | 25-60 | 1.77 (5) | 0.58 (3) | 3.1 (0.74-13.09); p=0.12 | 5.99 (0.66-54.59); p=0.11 |
| **33C** | 0-60 | 0.88 (5) | 0.44 (5) | 2 (0.58-6.92); p=0.28 | 2.56 (0.31-21.23); p = 0.38 |
|  | 0-24 | 0.35 (1) | 0.16 (1) | 2.14 (0.13-34.26); p=0.59 | 1155454 (0-.); p=0.996 |
|  | 25-60 | 1.42 (4) | 0.77 (4) | 1.85 (0.46-7.46); p=0.39 | 1.56 (0.15-16.52); p=0.71 |
| **34** | 0-60 | 2.45 (14) | 2.38 (27) | 1.03 (0.54-1.98); p=0.93 | 1.56 (0.58-4019); p = 0.38 |
|  | 0-24 | 1.73 (5) | 2.11 (13) | 0.82 (0.29-2.31); p=0.7 | 0.76 (0.17-3.44); p=0.72 |
|  | 25-60 | 3.19 (9) | 2.70 (14) | 1.19 (0.51-2.78); p=0.69 | 2.1 (0.54-8.24); p=0.29 |
| **35A/35C/42** | 0-60 | 1.40 (8) | 2.29 (26) | 0.61 (0.27-1.35); p=0.22 | 0.38 (0.15-0.96); p = 0.04 |
|  | 0-24 | 1.38 (4) | 2.60 (16) | 0.53 (0.17-1.59); p=0.26 | 0.35 (0.1-1.22); p=0.1 |
|  | 25-60 | 1.42 (4) | 1.93 (10) | 0.73 (0.23-2.36); p=0.6 | 0.37 (0.08-1.72); p=0.2 |
| **35B** | 0-60 | 2.28 (13) | 1.41 (16) | 1.63 (0.78-3.41); p=0.2 | 1.4 (0.53-3.68); p = 0.5 |
|  | 0-24 | 2.42 (7) | 0.97 (6) | 2.52 (0.84-7.58); p=0.1 | 5.18 (0.62-43.21); p=0.13 |
|  | 25-60 | 2.13 (6) | 1.93 (10) | 1.11 (0.4-3.08); p=0.85 | 0.57 (0.15-2.16); p=0.41 |
| **35F** | 0-60 | 1.05 (6) | 0.35 (4) | 3 (0.84-10.68); p=0.09 | 1.75 (0.34-8.97); p = 0.51 |
|  | 0-24 | 1.04 (3) | 0.16 (1) | 6.45 (0.67-62.29); p=0.11 | 2.02 (0.21-19.78); p=0.55 |
|  | 25-60 | 1.06 (3) | 0.58 (3) | 1.85 (0.37-9.22); p=0.45 | 1.98 (0.19-21.09); p=0.57 |
| **36** | 0-60 | 0.53 (3) | 0.26 (3) | 1.99 (0.4-9.91); p=0.4 | 17.62 (0.4-772.89); p = 0.14 |
|  | 0-24 | 0 (0) | 0 (0) | - | - |
|  | 25-60 | 1.06 (3) | 0.58 (3) | 1.85 (0.37-9.22); p=0.45 | 12.08 (0.31-467.95); p=0.18 |
| **41A** | 0-60 | 0.35 (2) | 0.35 (4) | 0.99 (0.18-5.44); p=0.99 | 4.66 (0.19-116.16); p = 0.35 |
|  | 0-24 | 0 (0) | 0.16 (1) | - | - |
|  | 25-60 | 0.71 (2) | 0.58 (3) | 1.23 (0.20-7.4); p=0.82 | 3.89 (0.07-206.77); p=0.5 |
| **43** | 0-60 | 0.53 (3) | 0.70 (8) | 0.74 (0.2-2.82); p=0.66 | 0.58 (0.12-2.73); p = 0.49 |
|  | 0-24 | 0.35 (1) | 0.81 (5) | 0.42 (0.05-3.65); p=0.44 | 0.53 (0.03-8.29); p=0.65 |
|  | 25-60 | 0.71 (2) | 0.58 (3) | 1.23 (0.20-7.4); p=0.82 | 0.62 (0.07-5.58); p=0.67 |
| **45** | 0-60 | 2.10 (12) | 1.85 (21) | 1.14 (0.56-2.33); p=0.72 | 3.41 (0.79-14.65); p = 0.1 |
|  | 0-24 | 1.04 (3) | 2.44 (15) | 0.42 (0.12-1.46); p=0.17 | 1.27 (0.13-12.62); p=0.84 |
|  | 25-60 | 3.19 (9) | 1.16 (6) | 2.82 (0.99-8.0); p=0.05 | 7.94 (1.14-55.3); p=0.04 |
| **46** | 0-60 | 0.35 (2) | 0.53 (6) | 0.66 (0.13-3.29); p=0.61 | 0.56 (0.07-4.27); p = 0.58 |
|  | 0-24 | 0.35 (1) | 0.65 (4) | 0.53 (0.06-4.77); p=0.57 | 0.39 (0.02-6.82); p=0.52 |
|  | 25-60 | 0.35 (1) | 0.39 (2) | 0.92 (0.08-10.19); p=0.95 | 1.25 (0.06-24.85); p=0.89 |
| **47A** | 0-60 | 1.93 (11) | 1.32 (15) | 1.47 (0.67-3.21); p=0.34 | 1.26 (0.44-3.62); p = 0.67 |
|  | 0-24 | 1.38 (4) | 0.32 (2) | 4.31 (0.78-23.66); p=0.09 | 43.74 (0.01-210690.1); p=0.38 |
|  | 25-60 | 2.48 (7) | 2.50 (13) | 0.99 (0.39-2.51); p=0.98 | 0.83 (0.24-2.87); p=0.77 |
| **47F** | 0-60 | 0 (0) | 0.09 (1) | p=0.48 | - |
|  | 0-24 | 0 (0) | 0 (0) | - | - |
|  | 25-60 | 0 (0) | 0.19 (1) | - | - |
| **48** | 0-60 | 0.88 (5) | 0.26 (3) | 3.33 (0.79-14); p=0.1 | 12.25 (0.37-409.43); p = 0.16 |
|  | 0-24 | 1.04 (3) | 0.16 (1) | 6.45 (0.67-62.29); p=0.11 | 121.81 (0-232000000); p=0.52 |
|  | 25-60 | 0.71 (2) | 0.39 (2) | 1.85 (0.26-13.18); p=0.54 | 10.53 (0.15-744.76); p=0.28 |
| **9like** | 0-60 | 0.88 (5) | 0.7 (8) | 1.24 (0.41-3.82); p=0.7 | 2.1 (0.38-11.5); p=0.40 |
|  | 0-24 | 1,73 (5) | 0,65 (4) | 2.69 (0.72-10.2); p=0.14 | 20.9 (0.54-803.82); p=0.1 |
|  | 25-60 | 0 (0) | 0,77 (4) | - | - |
| **15like** | 0-60 | 5.08 (29) | 6.08 (69) | 0.83 (0.53-1.29); p=0.4 | 0.95 (0.51-1.75); p=0.86 |
|  | 0-24 | 4,5 (13) | 6,33 (39) | 0.69 (0.37-1.33); p=0.27 | 0.76 (0.33-1.75); p=0.52 |
|  | 25-60 | 5,67 (16) | 5,78 (30) | 0.98 (0.52-1.83); p=0.95 | 1.06 (0.4-2.79); p=0.9 |
| The total number of children 0-60 months in Period-1: N=1135; and Period-2: N=571; children 0-24 months in Period-1: N=616 and Period-2: N=289; and children 25-60 months in Period-1: N=519 and in Period-2: N=282. OR: Odds Ratio; aOR: adjusted Odds Ratio, calculated using logistic regression; adjusted for breastfeeding status, HIV infection, antibiotic usage, co-trimoxazole prophylaxis, and tuberculosis treatment. Overall pneumococcus includes all samples positive for pneumococcal reference genes. PCV13-VT: vaccine serotypes including serotypes/groups 1, 3, 4, 5, 6A, 6B, 7A/F, 9A/V, 14, 18C, 19A, 19F and 23F. NVT: serotypes/serogroups not included in PCV13. NT: non-typeable pneumococci. | | | | | |
