# Supplementary material for: Streptococcus pneumoniae and other bacterial nasopharyngeal colonization seven years post-introduction of 13-valent pneumococcal conjugate vaccine in South African children
Source: Int J Infect Dis. 2023 Sep;134:45–52. doi: 10.1016/j.ijid.2023.05.016 (PMC10404162; doi:10.1016/j.ijid.2023.05.016)
Supplement: Supplementary file 10 [file mmc10.docx]

**Supplementary Table 3**: Carriage density of *Streptococcus pneumoniae* from Nasopharyngeal Swab samples collected from children 0-60 months of age in Period 1 (2010, n=1135) and Period 2 (2018, n=572).

| **Serotype/**  **group** |  | **0-60 months** | | | **0-24 months** | | | **25-60 months** | | |
| --- | --- | --- | --- | --- | --- | --- | --- | --- | --- | --- |
|  | **Period** | **n^†^** | **GMD (95% CI) ^‡^ log_10_ GE/ml** | **p-value** | **n^†^** | **GMD (95% CI) ^‡^ log_10_ GE/ml** | **p-value** | **n^†^** | **GMD (95% CI) ^‡^ log_10_ GE/ml** | **p-value** |
| **Overall** | 1 | 773 | 4.32 (4.23-4.41) |  | 407 | 4.46 (4.34-4.59) |  | 366 | 4.17 (4.04-4.30) |  |
|  | 2 | 282 | 4.50 (4.39-4.61) | 0.010 | 147 | 4.61 (4.45-4.77) | 0.247 | 135 | 4.38 (4.23-4.54) | 0.025 |
| **NVT** | 1 | 472 | 3.85 (3.68-4.03) |  | 245 | 4.03 (3.77-4.29) |  | 227 | 3.67 (3.45-3.91) |  |
|  | 2 | 207 | 4.67 (4.34-5.02) | 0.089 | 106 | 4.95 (4.48-5.48) | 0.070 | 101 | 4.38 (3.93-4.88) | 0.317 |
| **VT** | 1 | 464 | 4.32 (4.16-4.50) |  | 243 | 4.47 (4.24-4.71) |  | 221 | 4.17 (3.93-4.42) |  |
|  | 2 | 106 | 3.92 (3.61-4.26) | 0.000 | 57 | 3.95 (3.55-4.40) | 0.001 | 49 | 3.89 (3.40-4.45) | 0.000 |
| **NTSP** | 1 | 70 | 3.21 (2.91-3.54) |  | 36 | 3.49 (2.99-4.06) |  | 34 | 2.94 (2.60-3.33) |  |
|  | 2 | 23 | 3.95 (3.52-4.44) | 0.692 | 15 | 4.28 (3.68-4.98) | 0.117 | 8 | 3.40 (2.89-4.00) | 0.247 |
| **1** | 1 | 6 | 2.38 (1.20-4.73) |  | 6 | 2.38 (1.20-4.73) |  | 4 | 2.21 (0.70-6.98) |  |
|  | 2 | 0 | - | 0.040 | 0 | - | 0.097 | 0 | - | 0.191 |
| **3** | 1 | 24 | 2.16 (1.81-2.58) |  | 24 | 2.16 (1.81-2.58) |  | 15 | 2.10 (1.61-2.75) |  |
|  | 2 | 11 | 2.26 (1.71-3.00) | 0.217 | 11 | 2.26 (1.71-3.00) | 0.323 | 7 | 2.23 (1.53-3.25) | 0.529 |
| **4** | 1 | 16 | 3.13 (2.65-3.70) |  | 16 | 3.13 (2.65-3.70) |  | 11 | 3.39 (2.74-4.19) |  |
|  | 2 | 5 | 2.06 (1.65-2.57) | 0.143 | 5 | 2.06 (1.65-2.57) | 0.804 | 3 | 2.18 (1.86-2.54) | 0.112 |
| **5** | 1 | 39 | 1.79 (1.59-2.00) |  | 39 | 1.79 (1.59-2.00) |  | 17 | 1.63 (1.40-1.90) |  |
|  | 2 | 13 | 2.10 (1.54-2.85) | 0.615 | 13 | 2.10 (1.54-2.85) | 0.776 | 6 | 1.99 (1.02-3.91) | 0.984 |
| **6A** | 1 | 80 | 5.11 (4.85-5.39) |  | 80 | 5.11 (4.85-5.39) |  | 35 | 5.00 (4.58-5.45) |  |
|  | 2 | 9 | 4.29 (3.42-5.38) | 0.000 | 9 | 4.29 (3.42-5.38) | 0.056 | 2 | 4.05 (0.47-34.71) | 0.002 |
| **6B** | 1 | 87 | 3.83 (3.59-4.10) |  | 87 | 3.83 (3.59-4.10) |  | 41 | 3.83 (3.48-4.21) |  |
|  | 2 | 9 | 3.41 (2.76-4.22) | 0.002 | 9 | 3.41 (2.76-4.22) | 0.055 | 5 | 3.37 (2.24-5.06) | 0.010 |
| **7A/7F** | 1 | 2 | 2.01 (0.75-5.38) |  | 2 | 2.01 (0.75-5.38) |  | 1 | 2.18 |  |
|  | 2 | 2 | 2.46 (0.17-36.56) | 0.706 | 2 | 2.46 (0.17-36.56) | 0.966 | 1 | 1.99 | 0.501 |
| **9A/9V** | 1 | 21 | 2.94 (2.43-3.57) |  | 21 | 2.94 (2.43-3.57) |  | 14 | 2.58 (2.05-3.25) |  |
|  | 2 | 0 | - | 0.099 | 0 | - | 0.052 | 0 | - | 0.476 |
| **14** | 1 | 43 | 3.67 (3.37-4.00) |  | 43 | 3.67 (3.37-4.00) |  | 20 | 3.40 (2.97-3.89) |  |
|  | 2 | 8 | 3.95 (3.52-4.42) | 0.060 | 8 | 3.95 (3.52-4.42) | 0.303 | 3 | 3.71 (3.01-4.57) | 0.209 |
| **18C** | 1 | 6 | 3.64 (2.61-5.08) |  | 6 | 3.64 (2.61-5.08) |  | 3 | 3.49 (1.56-7.83) |  |
|  | 2 | 0 | - | 0.565 | 0 | - | - | 0 | - | 0.353 |
| **19A** | 1 | 59 | 3.32 (3.11-3.53) |  | 59 | 3.32 (3.11-3.53) |  | 28 | 3.05 (2.74-3.39) |  |
|  | 2 | 9 | 3.70 (3.08-4.45) | 0.003 | 9 | 3.70 (3.08-4.45) | 0.023 | 1 | 4.45 | 0.092 |
| **19F** | 1 | 75 | 4.85 (4.66-5.05) |  | 75 | 4.85 (4.66-5.05) |  | 28 | 4.69 (4.45-4.94) |  |
|  | 2 | 46 | 4.59 (4.41-4.78) | 0.039 | 46 | 4.59 (4.41-4.78) | 0.739 | 26 | 4.66 (4.44-4.90) | 0.009 |
| **23F** | 1 | 99 | 3.82 (3.62-4.03) |  | 99 | 3.82 (3.62-4.03) |  | 53 | 3.80 (3.54-4.08) |  |
|  | 2 | 6 | 4.16 (2.98-5.80) | 0.005 | 6 | 4.16 (2.98-5.80) | 0.091 | 1 | 5.49 | 0.014 |
| **6D** | 1 | 0 | - |  | 0 | - |  |  | - |  |
|  | 2 | 0 | - | - | 0 | - | - |  | - | - |
| **7B/7C/40** | 1 | 16 | 3.52 (2.97-4.18) |  | 7 | 3.74 (2.72-5.15) |  | 9 | 3.36 (2.64-4.28) |  |
|  | 2 | 5 | 3.51 (2.74-4.50) | 0.726 | 3 | 3.72 (2.04-6.77) | 0.220 | 2 | 3.22 (0.96-10.81) | 0.445 |
| **8** | 1 | 1 | 4.39 |  | 1 | 4.39 |  | 0 | - |  |
|  | 2 | 3 | 3.43 (1.52-7.77) | 0.230 | 3 | 3.43 (1.52-7.77) | 0.178 | 0 | - | - |
| **9-like** | 1 | 8 | 1.46 (1.23-1.73) |  | 4 | 1.38 (1.04-1.83) |  | 4 | 1.54 (1.05-2.27) |  |
|  | 2 | 5 | 1.41 (1.10-1.81) | 0.516 | 5 | 1.41 (1.10-1.81) | 0.064 | 0 | - | 0.148 |
| **9L/9N** | 1 | 10 | 3.67 (2.75-4.91) |  | 4 | 3.25 (1.28-8.24) |  | 6 | 3.99 (3.02-5.26) |  |
|  | 2 | 5 | 4.42 (2.14-9.12) | 0.706 | 4 | 4.18 (1.46-11.96) | 0.159 | 1 | 5.49 | 0.431 |
| **10A** | 1 | 11 | 2.78 (1.93-4.02) |  | 5 | 2.24 (0.93-5.38) |  | 6 | 3.34 (2.35-4.75) |  |
|  | 2 | 5 | 3.91 (2.76-5.54) | 0.882 | 3 | 4.19 (1.94-9.03) | 0.831 | 2 | 3.52 (0.25-50.59) | 0.883 |
| **10B** | 1 | 3 | 1.39 (0.86-2.24) |  | 3 | 1.39 (0.86-2.24) |  | 0 | - |  |
|  | 2 | 1 | 1.38 | 0.189 | 0 | - | 0.098 | 1 | 1.38 | 0.666 |
| **10C/10F** | 1 | 7 | 2.66 (2.15-3.30) |  | 2 | 2.87 (0.07-126.70) |  | 5 | 2.59 (2.07-3.23) |  |
|  | 2 | 3 | 2.65 (1.72-4.07) | 0.657 | 2 | 2.89 (1.04-8.07) | 0.182 | 1 | 2.22 | 0.085 |
|  |  |  |  |  |  |  |  |  |  |  |
| **11A/11D** | 1 | 28 | 4.19 (3.83-4.58) |  | 16 | 4.55 (4.08-5.08) |  | 12 | 3.75 (3.26-4.31) |  |
|  | 2 | 16 | 4.08 (3.53-4.72) | 0.839 | 7 | 4.30 (3.65-5.06) | 0.394 | 9 | 3.92 (3.02-5.08) | 0.336 |
| **11B/11C** | 1 | 3 | 3.54 (1.58-7.93) |  | 1 | 4.76 |  | 2 | 3.05 (0.24-37.96) |  |
|  | 2 | 1 | 2.13 | 0.466 | 1 | 2.13 | 0.344 | 0 | - | 0.868 |
| **11F** | 1 | 1 | 2.40 |  | 1 | 2.40 |  | 0 | - |  |
|  | 2 | 0 | - | - | 0 | - | - | 0 | - | - |
| **12A/12F/44** | 1 | 3 | 3.39 (1.72-6.67) |  | 3 | 3.39 (1.72-6.67) |  | 0 | - |  |
|  | 2 | 2 | 3.77 (0.76-18.70) | 0.751 | 1 | 4.28 | 0.881 | 1 | 3.33 | 0.133 |
| **12B** | 1 | 6 | 2.99 (2.33-3.84) |  | 4 | 3.16 (2.12-4.71) |  | 2 | 2.69 (0.30-24.30) |  |
|  | 2 | 2 | 3.47 (0.61-19.77) | 0.874 | 1 | 3.98 | 0.936 | 1 | 3.03 | 0.916 |
| **13** | 1 | 19 | 3.03 (2.45-3.75) |  | 12 | 3.04 (2.23-4.16) |  | 7 | 3.01 (2.12-4.27) |  |
|  | 2 | 13 | 3.45 (2.71-4.40) | 0.757 | 7 | 3.47 (2.39-5.04) | 0.704 | 6 | 3.43 (2.17-5.42) | 0.739 |
| **15A/15F** | 1 | 14 | 3.56 (3.16-4.01) |  | 8 | 3.65 (2.93-4.56) |  | 6 | 3.45 (3.10-3.83) |  |
|  | 2 | 16 | 3.95 (3.46-4.51) | 0.008 | 10 | 4.00 (3.37-4.75) | 0.041 | 6 | 3.86 (2.87-5.20) | 0.039 |
| **15B/15C** | 1 | 53 | 4.13 (3.82-4.48) |  | 25 | 4.52 (4.10-4.98) |  | 28 | 3.82 (3.38-4.31) |  |
|  | 2 | 10 | 3.83 (3.29-4.47) | 0.017 | 4 | 3.37 (2.62-4.35) | 0.024 | 6 | 4.17 (3.33-5.23) | 0.186 |
| **15-like** | 1 | 69 | 1.73 (1.60-1.87) |  | 39 | 1.72 (1.54-1.93) |  | 30 | 1.73 (1.54-1.93) |  |
|  | 2 | 29 | 1.83 (1.56-2.14) | 0.898 | 13 | 1.95 (1.50-2.54) | 0.550 | 16 | 1.74 (1.40-2.15) | 0.744 |
| **16A** | 1 | 4 | 1.65 (1.20-2.29) |  | 2 | 1.85 (0.16-21.58) |  | 2 | 1.48 (1.32-1.65) |  |
|  | 2 | 6 | 1.52 (1.08-2.13) | 0.118 | 3 | 1.64 (0.57-4.73) | 0.266 | 3 | 1.40 (0.76-2.59) | 0.230 |
| **16F** | 1 | 45 | 3.89 (3.57-4.24) |  | 23 | 4.09 (3.67-4.55) |  | 22 | 3.69 (3.21-4.25) |  |
|  | 2 | 13 | 3.63 (3.21-4.10) | 0.714 | 5 | 3.43 (2.81-4.18) | 0.272 | 8 | 3.76 (3.11-4.56) | 0.757 |
| **17F** | 1 | 28 | 2.85 (2.30-3.52) |  | 17 | 3.16 (2.41-4.16) |  | 11 | 2.42 (1.66-3.52) |  |
|  | 2 | 17 | 2.78 (2.23-3.47) | 0.677 | 10 | 2.77 (1.96-3.91) | 0.590 | 7 | 2.80 (1.98-3.96) | 0.891 |
| **31** | 1 | 9 | 2.65 (1.76-4.00) |  | 6 | 2.42 (1.30-4.52) |  | 3 | 3.18 (1.11-9.08) |  |
|  | 2 | 6 | 1.70 (0.87-3.32) | 0.068 | 4 | 1.74 (0.55-5.52) | 0.454 | 2 | 1.62 (0.00-644.70) | 0.593 |
| **32A/32F** | 1 | 4 | 1.58 (1.29-1.95) |  | 3 | 1.69 (1.64-1.74) |  | 1 | 1.30 |  |
|  | 2 | 1 | 2.67 | 0.984 | 0 | - | 0.908 | 1 | 2.67 | 0.157 |
| **33A/33F** | 1 | 9 | 1.84 (1.29-2.62) |  | 6 | 1.74 (1.12-2.68) |  | 3 | 2.05 (0.44-9.64) |  |
|  | 2 | 3 | 1.55 (0.41-5.83) | 0.424 | 2 | 1.14 (0.94-1.38) | 0.846 | 1 | 2.87 | 0.814 |
| **33B** | 1 | 7 | 1.95 (1.62-2.34) |  | 4 | 1.98 (1.36-2.87) |  | 3 | 1.91 (1.18-3.08) |  |
|  | 2 | 8 | 1.50 (1.28-1.75) | 0.947 | 3 | 1.60 (0.94-2.70) | 0.341 | 5 | 1.44 (1.15-1.82) | 0.198 |
| **33C** | 1 | 5 | 1.52 (1.34-1.72) |  | 1 | 1.57 |  | 4 | 1.50 (1.26-1.80) |  |
|  | 2 | 5 | 1.59 (1.02-2.50) | 0.144 | 1 | 1.18 | 0.753 | 4 | 1.72 (0.95-3.10) | 0.436 |
| **34** | 1 | 27 | 3.14 (2.81-3.52) |  | 13 | 2.96 (2.52-3.47) |  | 14 | 3.33 (2.79-3.97) |  |
|  | 2 | 14 | 3.47 (2.97-4.05) | 0.305 | 5 | 3.97 (3.15-5.01) | 0.897 | 9 | 3.22 (2.59-4.00) | 0.172 |
| **35A/35C/42** | 1 | 26 | 2.71 (2.23-3.30) |  | 16 | 3.18 (2.55-3.96) |  | 10 | 2.10 (1.46-3.03) |  |
|  | 2 | 8 | 1.85 (1.37-2.48) | 0.257 | 4 | 1.88 (1.05-3.35) | 0.061 | 4 | 1.82 (0.97-3.41) | 0.315 |
| **35B** | 1 | 16 | 3.29 (2.60-4.18) |  | 6 | 3.17 (2.19-4.58) |  | 10 | 3.37 (2.34-4.86) |  |
|  | 2 | 13 | 4.12 (3.76-4.51) | 0.014 | 7 | 4.15 (3.59-4.79) | 0.070 | 6 | 4.09 (3.46-4.83) | 0.368 |
| **35F** | 1 | 4 | 2.63 (1.56-4.43) |  | 1 | 4.28 |  | 3 | 2.23 (1.94-2.57) |  |
|  | 2 | 6 | 2.64 (2.17-3.20) | 0.521 | 3 | 2.69 (1.32-5.47) | 0.614 | 3 | 2.59 (2.30-2.91) | 0.614 |
| **36** | 1 | 3 | 1.92 (1.27-2.90) |  | 0 | - |  | 3 | 1.92 (1.27-2.90) |  |
|  | 2 | 3 | 1.36 (1.21-1.51) | 0.605 | 0 | - | - | 3 | 1.36 (1.21-1.51) | 0.605 |
| **37** | 1 | 0 | - |  | 0 | - |  | 0 | - |  |
|  | 2 | 0 | - | - | 0 | - | - | 0 | - | - |
| **41A** | 1 | 4 | 1.21 (0.92-1.60) |  | 1 | 1.24 |  | 3 | 1.20 (0.71-2.05) |  |
|  | 2 | 2 | 1.63 (0.42-6.26) | 0.403 | 0 | - | 0.988 | 2 | 1.63 (0.42-6.26) | 0.436 |
| **43** | 1 | 8 | 2.35 (1.97-2.81) |  | 5 | 2.60 (2.16-3.13) |  | 3 | 1.98 (1.17-3.36) |  |
|  | 2 | 3 | 2.96 (1.02-8.58) | 0.714 | 1 | 4.84 | 0.937 | 2 | 2.32 (1.21-4.45) | 0.539 |
| **45** | 1 | 21 | 1.78 (1.55-2.04) |  | 15 | 1.70 (1.44-2.02) |  | 6 | 1.98 (1.46-2.70) |  |
|  | 2 | 12 | 1.81 (1.55-2.11) | 0.089 | 3 | 1.73 (0.99-3.01) | 0.908 | 9 | 1.84 (1.50-2.25) | 0.021 |
| **46** | 1 | 6 | 3.03 (2.38-3.87) |  | 4 | 3.18 (2.13-4.75) |  | 2 | 2.76 (0.34-22.25) |  |
|  | 2 | 2 | 3.47 (0.61-19.77) | 0.869 | 1 | 3.98 | 0.937 | 1 | 3.03 | 0.908 |
|  |  |  |  |  |  |  |  |  |  |  |
| **47A** | 1 | 15 | 1.35 (1.20-1.52) |  | 2 | 1.28 (1.03-1.61) |  | 13 | 1.37 (1.19-1.57) |  |
|  | 2 | 11 | 1.90 (1.37-2.62) | 0.192 | 4 | 1.86 (1.15-3.00) | 0.163 | 7 | 1.92 (1.12-3.28) | 0.396 |
| **47F** | 1 | 1 | 1.81 |  | 1 | 1.59 |  | 1 | 1.81 |  |
|  | 2 | 0 | - | 0.118 | 0 | - | - | 0 | - | - |
| **48** | 1 | 3 | 1.68 (1.44-1.96) |  | 0 | - |  | 2 | 1.73 (1.02-2.91) |  |
|  | 2 | 5 | 1.86 (1.29-2.68) | 0.692 | 3 | 1.63 (1.09-2.43) | 0.248 | 2 | 2.27 (0.06-87.79) | 0.224 |
| The total number of children 0-60 months in Period-1: N=1135; and Period-2: N=571; children 0-24 months in Period-1: N=616 and Period-2: N=289; and children 25-60 months in Period-1: N=519 and in Period-2: N=282. **^†^**n is the number of isolates. ^‡^ GMD is the Geometric Mean Density (95% Confidence Interval). p-values were considered significant if ≤0.01. Density of carriage was determined through quantitative real-time nanofluidic PCR in the Fluidigm. | | | | | | | | | | |
