# Supplementary material for: Streptococcus pneumoniae and other bacterial nasopharyngeal colonization seven years post-introduction of 13-valent pneumococcal conjugate vaccine in South African children
Source: Int J Infect Dis. 2023 Sep;134:45–52. doi: 10.1016/j.ijid.2023.05.016 (PMC10404162; doi:10.1016/j.ijid.2023.05.016)
Supplement: Supplementary file 11 [file mmc11.docx]

|  | **Period-2  % (n); N=571** | **Period-1  % (n); N=1135** | **OR (95% CI)**  **p-value** | **aOR (95% CI)**  **P-value** |
| --- | --- | --- | --- | --- |
| ***A. Baumannii*** | 6 (34) | 4.6 (52) | 1.32(0.85-2.06); p=0.22 | 0.84(0.49-1.46); p=0.539 |
| ***B. Holmesii*** | 0.2 (1) | 0.4 (4) | 0.5(0.06-4.45); p=0.53 | 0.36(0.03-4.17); p=0.41 |
| ***B. Parapertussis/bronchiseptica*** | 0.2 (1) | 0.2 (2) | 0.99(0.09-10.98); p=0.99 | 0.18(0.02-2.23); p=0.18 |
| ***H. influenzae*** | 0.7 (4) | 1.1 (12) | 0.66(0.21-2.06); p=0.47 | 0.46(0.13-1.66); p=0.24 |
| ***- H. influenzae-b*** | 0 (0) | 0.62 (7) | p=0.06 | - |
| **- *NTHI*** | 49 (280) | 57.1 (648) | 0.72(0.59-0.88); p<0.001 | 0.86(0.65-1.13); p=0.27 |
| ***K. pneumoniae*** | 14.2 (81) | 6.7 (76) | 2.3(1.65-3.21); p<0.001 | 1.69(1.07-2.66); p=0.02 |
| ***M. catarrhalis*** | 57.4 (328) | 63.3 (719) | 0.78(0.64-0.96); p=0.02 | 0.8(0.6-1.06); p=0.11 |
| ***N. Lactamica*** | 8.9 (51) | 6.8 (77) | 1.35(0.93-1.95); p=0.11 | 2.39(1.29-4.43); p=0.006 |
| ***N. meningitidis*** | 0.5 (3) | 0.9 (10) | 0.59(0.16-2.17); p=0.43 | 0.38(0.08-1.76); p=0.22 |
| ***S. aureus*** | 5.1 (29) | 8.8 (100) | 0.55(0.36-0.85); p=0.01 | 0.67(0.38-1.17); p=0.16 |
| ***S. pneumoniae*** | 49.4 (282) | 68.1 (773) | 0.46(0.37-0.56); p<0.001 | 0.66 (0.54-0.88); p=0.004 |
| ***S. oralis*** | 17.3 (99) | 17.8 (202) | 0.97(0.74-1.26); p=0.81 | 0.9(0.63-1.27); p=0.54 |
| ***S. pyogenes*** | 1.2 (7) | 3.3 (37) | 0.37(0.16-0.83); p=0.02 | 0.79(0.28-2.24); p=0.65 |
| The total number of children 0-60 months in Period-1: N=1135; and Period-2: N=571; OR: Odds Ratio; aOR: adjusted Odds Ratio, calculated using logistic regression; adjusted for breastfeeding status, HIV infection, antibiotic usage, co-trimoxazole prophylaxis, and tuberculosis treatment. | | | | |

**Supplementary Table 4:** Prevalence of bacterial colonisation from Nasopharyngeal Swab samples in Period-1 (2010) and Period-2 (2018) in Sowetan children 0-60 months of age.
