# Supplementary material for: Streptococcus pneumoniae and other bacterial nasopharyngeal colonization seven years post-introduction of 13-valent pneumococcal conjugate vaccine in South African children
Source: Int J Infect Dis. 2023 Sep;134:45–52. doi: 10.1016/j.ijid.2023.05.016 (PMC10404162; doi:10.1016/j.ijid.2023.05.016)
Supplement: Supplementary file 12 [file mmc12.docx]

**Supplementary Table 5**: Bacterial carriage density from Nasopharyngeal Swab samples collected from children 0-60 months of age in Period 1 (2010, n=1135) and Period 2 (2018, n=572).

| **Bacterial species** |  | **All Ages** | | | **Under 24 months** | | | **Over 24 months** | | |
| --- | --- | --- | --- | --- | --- | --- | --- | --- | --- | --- |
|  | **P^*^** | **n^†^** | **GMD (95% CI) ^‡^ log_10_ GE/ml** | **p-value** | **n^†^** | **GMD (95% CI) ^‡^ log_10_ GE/ml** | **p-value** | **n^†^** | **GMD (95% CI) ^‡^ log_10_ GE/ml** | **p-value** |
| ***Acinetobacter baumannii*** | 1 | 52 | 1.88 (1.75-2.02) |  | 27 | 2.16 (1.96-2.38) |  | 25 | 1.62 (1.50-1.75) |  |
|  | 2 | 34 | 1.53 (1.43-1.64) | 0.273 | 19 | 1.53 (1.41-1.66) | 0.747 | 15 | 1.52 (1.34-1.73) | 0.048 |
| ***Bordetella holmesii*** | 1 | 4 | 5.49 (3.80-7.94) |  | 2 | 5.29 (0.60-47.01) |  | 2 | 5.70 (0.35-92.77) |  |
|  | 2 | 1 | 5.62 | 0.356 | 0 | - | 0.054 | 1 | 5.62 | 0.644 |
| ***Bordetella parapertussis/bronchiseptica*** | 1 | 2 | 3.86 (0.61-24.33) |  | 2 | 3.86 (0.61-24.33) |  | 0 | - |  |
|  | 2 | 1 | 5.34 | 0.207 | 0 | - | 0.040 | 1 | 5.34 - | 0.666 |
| ***Haemophilus influenzae*** | 1 | 12 | 4.56 (3.77-5.52) |  | 6 | 5.06 (4.29-5.96) |  | 6 | 4.11 (2.74-6.17) |  |
|  | 2 | 4 | 4.81 (3.42-6.75) | 0.236 | 1 | 3.50 | 0.028 | 3 | 5.34 (4.87-5.86) | 0.489 |
| ***Haemophilus influenzae type b*** | 1 | 7 | 4.64 (3.79-5.68) |  | 3 | 5.33 (2.86-9.93) |  | 4 | 4.18 (3.32-5.26) |  |
|  | 2 | 0 | - | 0.133 | 0 | - | 0.402 | 0 | - | 0.231 |
| ***Non-typeable Haemophilus influenzae*** | 1 | 648 | 3.07 (2.97-3.18) |  | 332 | 3.12 (2.96-3.29) |  | 316 | 3.03 (2.89-3.17) |  |
|  | 2 | 280 | 2.94 (2.79-3.10) | 0.133 | 135 | 3.09 (2.88-3.32) | 0.158 | 145 | 2.81 (2.61-3.04) | 0.350 |
| ***Klebsiella pneumoniae*** | 1 | 76 | 2.24 (2.06-2.44) |  | 46 | 2.32 (2.06-2.62) |  | 30 | 2.13 (1.89-2.41) |  |
|  | 2 | 80 | 2.16 (2.01-2.31) | 0.019 | 38 | 2.11 (1.89-2.36) | 0.075 | 42 | 2.20 (2.02-2.40) | 0.086 |
| ***Moraxella catarrhalis*** | 1 | 719 | 4.15 (4.05-4.25) |  | 388 | 4.18 (4.03-4.32) |  | 331 | 4.12 (3.98-4.25) |  |
|  | 2 | 329 | 4.23 (4.09-4.38) | 0.429 | 174 | 4.39 (4.20-4.60) | 0.720 | 155 | 4.06 (3.84-4.29) | 0.582 |
| ***Neisseria lactamica*** | 1 | 77 | 2.35 (2.17-2.54) |  | 49 | 2.31 (2.07-2.57) |  | 28 | 2.42 (2.16-2.71) |  |
|  | 2 | 51 | 2.21 (2.00-2.43) | 0.023 | 27 | 2.32 (2.05-2.63) | 0.407 | 24 | 2.09 (1.78-2.44) | 0.005 |
| ***Neisseria meningitidis*** | 1 | 10 | 3.21 (2.79-3.70) |  | 4 | 3.12 (2.57-3.80) |  | 6 | 3.27 (2.53-4.23) |  |
|  | 2 | 3 | 2.46 (1.38-4.39) | 0.100 | 3 | 2.46 (1.38-4.39) | 0.624 | 0 | - | 0.005 |
| ***Staphylococcus aureus*** | 1 | 100 | 3.15 (2.92-3.40) |  | 67 | 3.17 (2.89-3.49) |  | 33 | 3.10 (2.70-3.55) |  |
|  | 2 | 29 | 2.95 (2.55-3.41) | 0.156 | 19 | 2.94 (2.43-3.56) | 0.247 | 10 | 2.95 (2.26-3.85) | 0.678 |
| ***Streptococcus pneumoniae*** | 1 | 773 | 4.32 (4.23-4.41) |  | 407 | 4.46 (4.34-4.59) |  | 366 | 4.17 (4.04-4.30) |  |
|  | 2 | 282 | 4.50 (4.39-4.61) | 0.010 | 147 | 4.61 (4.45-4.77) | 0.247 | 135 | 4.38 (4.23-4.54) | 0.025 |
| ***Streptococcus oralis*** | 1 | 202 | 2.52 (2.42-2.61) |  | 101 | 2.53 (2.38-2.68) |  | 101 | 2.50 (2.39-2.63) |  |
|  | 2 | 99 | 2.38 (2.26-2.50) | 0.401 | 38 | 2.29 (2.09-2.51) | 0.204 | 61 | 2.44 (2.29-2.59) | 0.815 |
| ***Streptococcus pyogenes*** | 1 | 37 | 2.26 (1.96-2.60) |  | 13 | 2.30 (1.81-2.91) |  | 24 | 2.23 (1.85-2.70) |  |
|  | 2 | 7 | 1.57 (1.17-2.13) | 0.542 | 2 | 1.90 (0.01-503.00) | 0.410 | 5 | 1.46 (1.15-1.86) | 0.226 |
| **^*^**P denotes the study Period. **^†^**n is the number of isolates. ^‡^ GMD is the Geometric Mean Density (95% Confidence Interval). p-values were considered significant if ≤0.01. Density of carriage was determined through quantitative real-time nanofluidic PCR in the Fluidigm. | | | | | | | | | | |
