# Supplementary material for: Streptococcus pneumoniae and other bacterial nasopharyngeal colonization seven years post-introduction of 13-valent pneumococcal conjugate vaccine in South African children
Source: Int J Infect Dis. 2023 Sep;134:45–52. doi: 10.1016/j.ijid.2023.05.016 (PMC10404162; doi:10.1016/j.ijid.2023.05.016)
Supplement: Supplementary file 13 [file mmc13.docx]

**Supplementary Table 6**: Hierarchy of co-colonising pneumococcal serotypes detected in Nasopharyngeal Swab samples collected from Sowetan children 0-60 months-of-age in Period-1 (2010, n=1135) and Period-2 (2018, n=572).

| **Serotype/ group** | **P^*^** | **Primary Coloniser  % (n/N) ^†^** | **Co-Colonisation  % (n/N) ^†^** | **Second Serotype  % (n/N) ^†^** | **Third Serotype  % (n/N) ^†^** | **≥ Four Serotypes  % (n/N) ^†^** |
| --- | --- | --- | --- | --- | --- | --- |
| **VT** | 1 | 84.3% (391/464) | 15.7% (73/464) | - | - | - |
|  | 2 | 73.6% (78/106) | 26.4% (28/106) | - | - | - |
| **NVT** | 1 | 70.1% (331/472) | 29.9% (141/472) | - | - | - |
|  | 2 | 87.0% (180/207) | 13.0% (27/207) | - | - | - |
| **NTSP** | 1 | 100% (70/70) | - | - | - | - |
|  | 2 | 100% (23/23) | - | - | - | - |
| **1** | 1 | 33.3% (2/6) | 66.7% (4/6) | 33.3% (2/6) | 33.3% (2/6) | - |
|  | 2 | - | - | - | - | - |
| **3** | 1 | 37.5% (9/24) | 62.5% (15/24) | 45.8% (11/24) | 12.5% (3/24) | 4.2% (1/24) |
|  | 2 | 54.5% (6/11) | 45.5% (5/11) | 36.4% (4/11) | - | 9.1% (1/11) |
| **4** | 1 | 50.0% (8/16) | 50.0% (8/16) | 31.3% (5/16) | 12.5% (2/16) | 6.3% (1/16) |
|  | 2 | 40.0% (2/5) | 60.0% (3/5) | 60.0% (3/5) | - | - |
| **5** | 1 | 20.5% (8/39) | 79.5% (31/39) | 38.5% (15/39) | 23.1% (9/39) | 17.9% (7/39) |
|  | 2 | 23.1% (3/13) | 76.9% (10/13) | 38.5% (5/13) | 30.8% (4/13) | 7.7% (1/13) |
| **6A** | 1 | 92.5% (74/80) | 7.5% (6/80) | 7.5% (6/80) | - | - |
|  | 2 | 77.8% (7/9) | 22.2% (2/9) | - | 22.2% (2/9) | - |
| **6B** | 1 | 65.5% (57/87) | 34.5% (30/87) | 26.4% (23/87) | 6.9% (6/87) | 1.1% (1/87) |
|  | 2 | 55.6% (5/9) | 44.4% (4/9) | 44.4% (4/9) | - | - |
| **7A/7F** | 1 | 50.0% (1/2) | 50.0% (1/2) | 50.0% (1/2) | - | - |
|  | 2 | - | 100% (2/2) | 100% (2/2) | - | - |
| **9A/9V** | 1 | 42.9% (9/21) | 57.1% (12/21) | 42.9% (9/21) | 14.3% (3/21) | - |
|  | 2 | - | - | - | - | - |
| **14** | 1 | 69.8% (30/43) | 30.2% (13/43) | 27.9% (12/43) | 2.3% (1/43) | - |
|  | 2 | 62.5% (5/8) | 37.5% (3/8) | 25.0% (2/8) | 12.5% (1/8) | - |
| **18C** | 1 | 83.3% (5/6) | 16.7% (1/6) | 16.7% (1/6) | - | - |
|  | 2 | - | - | - | - | - |
| **19A** | 1 | 67.8% (40/59) | 32.2% (19/59) | 25.4% (15/59) | 5.1% (3/59) | 1.7% (1/59) |
|  | 2 | 44.4% (4/9) | 55.6% (5/9) | 22.2% (2/9) | - | 33.3% (3/9) |
| **19F** | 1 | 92.0% (69/75) | 8.0% (6/75) | 6.7% (5/75) | 1.3% (1/75) | - |
|  | 2 | 93.5% (43/46) | 6.5% (3/46) | 4.3% (2/46) | - | 2.2% (1/46) |
| **23F** | 1 | 82.8% (82/99) | 17.2% (17/99) | 14.1% (14/99) | 2.0% (2/99) | 1.0% (1/99) |
|  | 2 | 66.7% (4/6) | 33.3% (2/6) | 33.3% (2/6) | - | - |
| **2** | 1 | - | 100% (1/1) | 100% (1/1) | - | - |
|  | 2 | - | 100% (3/3) | 33.3% (1/3) | 33.3% (1/3) | 33.3% (1/3) |
| **6C** | 1 | 100% (5/5) | - | - | - | - |
|  | 2 | 86.7% (13/15) | 13.3% (2/15) | 13.3% (2/15) | - | - |
| **7B/7C/40** | 1 | 62.5% (10/16) | 37.5% (6/16) | 31.3% (5/16) | 6.3% (1/16) | - |
|  | 2 | 80.0% (4/5) | 20.0% (1/5) | 20.0% (1/5) | - | - |
| **8** | 1 | - | 100% (1/1) | 100% (1/1) | - | - |
|  | 2 | 66.7% (2/3) | 33.3% (1/3) | 33.3% (1/3) | - | - |
| **9like** | 1 | - | 100% (8/8) | 37.5% (3/8) | 50.0% (4/8) | 12.5% (1/8) |
|  | 2 | - | 100% (5/5) | 40.0% (2/5) | 40.0% (2/5) | 20.0% (1/5) |
| **9LN** | 1 | 50.0% (5/10) | 50.0% (5/10) | 40.0% (4/10) | 10.0% (1/10) | - |
|  | 2 | 80.0% (4/5) | 20.0% (1/5) | 20.0% (1/5) | - | - |
| **10A** | 1 | 72.7% (8/11) | 27.3% (3/11) | - | 9.1% (1/11) | 18.2% (2/11) |
|  | 2 | 80.0% (4/5) | 20.0% (1/5) | - | - | 20.0% (1/5) |
| **10B** | 1 | - | 100% (3/3) | 66.7% (2/3) | - | 33.3% (1/3) |
|  | 2 | - | 100% (1/1) | - | - | 100% (1/1) |
| **10C/10F** | 1 | 14.3% (1/7) | 85.7% (6/7) | 71.4% (5/7) | 14.3% (1/7) | - |
|  | 2 | 66.7% (2/3) | 33.3% (1/3) | 33.3% (1/3) | - | - |
| **11A/10D** | 1 | 78.6% (22/28) | 21.4% (6/28) | 17.9% (5/28) | 3.6% (1/28) | - |
|  | 2 | 68.8% (11/16) | 31.3% (5/16) | 12.5% (2/16) | 18.8% (3/16) | - |
| **11B/11C** | 1 | 66.7% (2/3) | 33.3% (1/3) | - | 33.3% (1/3) | - |
|  | 2 | - | 100% (1/1) | - | 100% (1/1) | - |
| **11F** | 1 | - | 100% (1/1) | - | 100% (1/1) | - |
|  | 2 | - | - | - | - | - |
| **12A/12F/44** | 1 | 66.7% (2/3) | 33.3% (1/3) | 33.3% (1/3) | - | - |
|  | 2 | 50.0% (1/2) | 50.0% (1/2) | 50.0% (1/2) | - | - |
| **12B** | 1 | - | 100% (6/6) | 66.7% (4/6) | 33.3% (2/6) | - |
|  | 2 | - | 100% (2/2) | 50.0% (1/2) | - | 50.0% (1/2) |
| **13** | 1 | 73.7% (14/19) | 26.3% (5/19) | 21.1% (4/19) | 5.3% (1/19) | - |
|  | 2 | 92.3% (12/13) | 7.7% (1/13) | 7.7% (1/13) | - | - |
| **15A/15F** | 1 | 57.1% (8/14) | 42.9% (6/14) | 35.7% (5/14) | 7.1% (1/14) | - |
|  | 2 | 87.5% (14/16) | 12.5% (2/16) | 12.5% (2/16) | - | - |
| **15B/15C** | 1 | 83.0% (44/53) | 17.0% (9/53) | 15.1% (8/53) | 1.9% (1/53) | - |
|  | 2 | 80.0% (8/10) | 20.0% (2/10) | 20.0% (2/10) | - | - |
| **15like** | 1 | 20.3% (14/69) | 79.7% (55/69) | 46.4% (32/69) | 26.1% (18/69) | 7.2% (5/69) |
|  | 2 | 37.9% (11/29) | 62.1% (18/29) | 41.4% (12/29) | 6.9% (2/29) | 13.8% (4/29) |
| **16A** | 1 | - | 100% (4/4) | - | 50.0% (2/4) | 50.0% (2/4) |
|  | 2 | - | 100% (6/6) | 33.3% (2/6) | 33.3% (2/6) | 33.3% (2/6) |
| **16F** | 1 | 77.8% (35/45) | 22.2% (10/45) | 20.0% (9/45) | 2.2% (1/45) | - |
|  | 2 | 92.3% (12/13) | 7.7% (1/13) | 7.7% (1/13) | - | - |
| **17F** | 1 | 50.0% (14/28) | 50.0% (14/28) | 32.1% (9/28) | 14.3% (4/28) | 3.6% (1/28) |
|  | 2 | 41.2% (7/17) | 58.8% (10/17) | 23.5% (4/17) | 11.8% (2/17) | 23.5% (4/17) |
| **18A** | 1 | 66.7% (2/3) | 33.3% (1/3) | - | 33.3% (1/3) | - |
|  | 2 | - | - | - | - | - |
| **18B** | 1 | 60.0% (3/5) | 40.0% (2/5) | 40.0% (2/5) | - | - |
|  | 2 | 100% (1/1) | - | - | - | - |
| **19B** | 1 | 25.0% (1/4) | 75.0% (3/4) | 25.0% (1/4) | 25.0% (1/4) | 25.0% (1/4) |
|  | 2 | 50.0% (2/4) | 50.0% (2/4) | 25.0% (1/4) | 25.0% (1/4) | - |
| **20** | 1 | 93.3% (14/15) | 6.7% (1/15) | - | 6.7% (1/15) | - |
|  | 2 | 75.0% (3/4) | 25.0% (1/4) | 25.0% (1/4) | - | - |
| **21** | 1 | 72.7% (8/11) | 27.3% (3/11) | 27.3% (3/11) | - | - |
|  | 2 | 46.2% (6/13) | 53.8% (7/13) | 38.5% (5/13) | 7.7% (1/13) | 7.7% (1/13) |
| **22A** | 1 | 43.8% (7/16) | 56.3% (9/16) | 37.5% (6/16) | 18.8% (3/16) | - |
|  | 2 | - | 100% (2/2) | 100% (2/2) | - | - |
| **22F** | 1 | 100% (6/6) | - | - | - | - |
|  | 2 | 100% (1/1) | - | - | - | - |
| **23A** | 1 | 29.6% (8/27) | 70.4% (19/27) | 63.0% (17/27) | 3.7% (1/27) | 3.7% (1/27) |
|  | 2 | 70.0% (7/10) | 30.0% (3/10) | 30.0% (3/10) | - | - |
| **23B** | 1 | 80.0% (16/20) | 20.0% (4/20) | 20.0% (4/20) | - | - |
|  | 2 | 95.2% (20/21) | 4.8% (1/21) | 4.8% (1/21) | - | - |
| **24A** | 1 | - | - | - | - | - |
|  | 2 | - | 100% (2/2) | 50.0% (1/2) | - | 50.0% (1/2) |
| **24B/24F** | 1 | - | - | - | - | - |
|  | 2 | 20.0% (1/5) | 80.0% (4/5) | - | 40.0% (2/5) | 40.0% (2/5) |
| **27** | 1 | 33.3% (1/3) | 66.7% (2/3) | 33.3% (1/3) | 33.3% (1/3) | - |
|  | 2 | - | 100% (4/4) | - | 25.0% (1/4) | 75.0% (3/4) |
| **29** | 1 | - | 100% (6/6) | 100% (6/6) | - | - |
|  | 2 | - | 100% (8/8) | 62.5% (5/8) | 12.5% (1/8) | 25.0% (2/8) |
| **31** | 1 | 66.7% (6/9) | 33.3% (3/9) | - | 33.3% (3/9) | - |
|  | 2 | 16.7% (1/6) | 83.3% (5/6) | - | 33.3% (2/6) | 50.0% (3/6) |
| **32A/32F** | 1 | - | 100% (4/4) | - | 25.0% (1/4) | 75.0% (3/4) |
|  | 2 | - | 100% (1/1) | - | - | - |
| **33A/33F** | 1 | 33.3% (3/9) | 66.7% (6/9) | - | - | 66.7% (6/9) |
|  | 2 | - | 100% (3/3) | - | 33.3% (1/3) | 66.7% (2/3) |
| **33B** | 1 | 14.3% (1/7) | 85.7% (6/7) | 57.1% (4/7) | 14.3% (1/7) | 14.3% (1/7) |
|  | 2 | 25.0% (2/8) | 75.0% (6/8) | 37.5% (3/8) | 12.5% (1/8) | 25.0% (2/8) |
| **33C** | 1 | - | 100% (5/5) | 20.0% (1/5) | 20.0% (1/5) | 60.0% (3/5) |
|  | 2 | - | 100% (5/5) | 20.0% (1/5) | 40.0% (2/5) | 40.0% (2/5) |
| **34** | 1 | 63.0% (17/27) | 37.0% (10/27) | 37.0% (10/27) | - | - |
|  | 2 | 78.6% (11/14) | 21.4% (3/14) | 14.3% (2/14) | 7.1% (1/14) | - |
| **35A/35C/42** | 1 | 46.2% (12/26) | 53.8% (14/26) | 26.9% (7/26) | 15.4% (4/26) | 11.5% (3/26) |
|  | 2 | - | 100% (8/8) | 50.0% (4/8) | 37.5% (3/8) | 12.5% (1/8) |
| **35B** | 1 | 50.0% (8/16) | 50.0% (8/16) | 43.8% (7/16) | 6.3% (1/16) | - |
|  | 2 | 84.6% (11/13) | 15.4% (2/13) | 7.7% (1/13) | - | 7.7% (1/13) |
| **35F** | 1 | 50.0% (2/4) | 50.0% (2/4) | 25.0% (1/4) | - | 25.0% (1/4) |
|  | 2 | 66.7% (4/6) | 33.3% (2/6) | 33.3% (2/6) | - | - |
| **36** | 1 | - | 100% (3/3) | 33.3% (1/3) | 66.7% (2/3) | - |
|  | 2 | - | 100% (3/3) | 33.3% (1/3) | - | 66.7% (2/3) |
| **41A** | 1 | - | 100% (4/4) | - | 25.0% (1/4) | 75.0% (3/4) |
|  | 2 | 50.0% (1/2) | 50.0% (1/2) | - | - | 50.0% (1/2) |
| **43** | 1 | 25.0% (2/8) | 75.0% (6/8) | 62.5% (5/8) | - | 12.5% (1/8) |
|  | 2 | 66.7% (2/3) | 33.3% (1/3) | 33.3% (1/3) | - | - |
| **45** | 1 | 19.0% (4/21) | 81.0% (17/21) | 52.4% (11/21) | 19.0% (4/21) | 9.5% (2/21) |
|  | 2 | - | 100% (12/12) | 41.7% (5/12) | 25.0% (3/12) | 33.3% (4/12) |
| **46** | 1 | 50.0% (3/6) | 50.0% (3/6) | 16.7% (1/6) | 16.7% (1/6) | 16.7% (1/6) |
|  | 2 | - | 100% (2/2) | - | 100% (2/2) | - |
| **47A** | 1 | 6.7% (1/15) | 93.3% (14/15) | 40.0% (6/15) | 26.7% (4/15) | 26.7% (4/15) |
|  | 2 | 18.2% (2/11) | 81.8% (9/11) | 36.4% (4/11) | 18.2% (2/11) | 27.3% (3/11) |
| **47F** | 1 | - | 100% (1/1) | 100% (1/1) | - | - |
|  | 2 | - | - | - | - | - |
| **48** | 1 | - | 100% (3/3) | - | - | 100% (3/3) |
|  | 2 | - | 100% (5/5) | 20.0% (1/5) | 20.0% (1/5) | 60.0% (3/5) |
| **^*^**P denotes the study Period. **^†^**n is the number of isolates in each category for each period and N is the total isolates identified as each serotype/group. The rank was determined according to colonization density. | | | | | | |
