# Supplementary material for: Streptococcus pneumoniae and other bacterial nasopharyngeal colonization seven years post-introduction of 13-valent pneumococcal conjugate vaccine in South African children
Source: Int J Infect Dis. 2023 Sep;134:45–52. doi: 10.1016/j.ijid.2023.05.016 (PMC10404162; doi:10.1016/j.ijid.2023.05.016)
Supplement: Supplementary file 14 [file mmc14.docx]

**Supplementary Table 7**: Multiple concurrent bacterial colonisers detected in Nasopharyngeal Swab samples collected from Sowetan children 0-60 months-of-age in Period-1 (2010, n=1135) and Period-2 (2018, n=572).

| **Bacteria** | **p** | **Primary Coloniser % (n/N) †** | **Co-Colonisation % (n/N) †** | **Second Serotype % (n/N) †** | **Third Serotype % (n/N) †** | **≥ Four Serotypes % (n/N) †** |
| --- | --- | --- | --- | --- | --- | --- |
| *A. baumannii* | 1 | 9.6% (5/52) | 90.4% (47/52) | 9.6% (5/52) | 28.8% (15/52) | 51.9% (27/52) |
|  | 2 | 11.8% (4/34) | 88.2% (30/34) | 29.4% (10/34) | 11.8% (4/34) | 47.1% (16/34) |
| *S. pneumoniae* | 1 | 58.2% (450/773) | 41.8% (323/773) | 33.1% (256/773) | 7.5% (58/773) | 1.2% (9/773) |
|  | 2 | 61.3% (173/282) | 38.7% (109/282) | 33.7% (95/282) | 4.6% (13/282) | 0.4% (1/282) |
| *B. holmesii* | 1 | 50.0% (2/4) | 50.0% (2/4) | 25.0% (1/4) | 25.0% (1/4) |  |
|  | 2 | 100% (1/1) |  |  |  |  |
| *B. parapertussis/ bronchiseptica* | 1 |  | 100% (2/2) | 50.0% (1/2) | 50.0% (1/2) |  |
|  | 2 | 100% (1/1) |  |  |  |  |
| *H. influenzae* | 1 | 41.7% (5/12) | 58.3% (7/12) | 41.7% (5/12) | 16.7% (2/12) |  |
|  | 2 | 25.0% (1/4) | 75.0% (3/4) | 75.0% (3/4) |  |  |
| *-H. influenzae. type b* | 1 | 28.6% (2/7) | 71.4% (5/7) | 71.4% (5/7) |  |  |
|  | 2 |  |  |  |  |  |
| *-NTHI* | 1 | 21.6% (140/648) | 78.4% (508/648) | 36.7% (238/648) | 35.8% (232/648) | 5.9% (38/648) |
|  | 2 | 23.5% (66/281) | 76.5% (215/281) | 36.7% (103/281) | 32.7% (92/281) | 7.1% (20/281) |
| *K. pneumoniae* | 1 | 15.8% (12/76) | 84.2% (64/76) | 25.0% (19/76) | 34.2% (26/76) | 25.0% (19/76) |
|  | 2 | 22.2% (18/81) | 77.8% (63/81) | 30.9% (25/81) | 21.0% (17/81) | 25.9% (21/81) |
| *M. catarrhalis* | 1 | 52.2% (375/719) | 47.8% (344/719) | 33.5% (241/719) | 12.1% (87/719) | 2.2% (16/719) |
|  | 2 | 61.1% (201/329) | 38.9% (128/329) | 27.7% (91/329) | 10.3% (34/329) | 0.9% (3/329) |
| *N. lactamica* | 1 | 3.9% (3/77) | 96.1% (74/77) | 13.0% (10/77) | 40.3% (31/77) | 42.9% (33/77) |
|  | 2 | 5.9% (3/51) | 94.1% (48/51) | 15.7% (8/51) | 35.3% (18/51) | 43.1% (22/51) |
| *N. meningitidis* | 1 | 10.0% (1/10) | 90.0% (9/10) | 20.0% (2/10) | 60.0% (6/10) | 10.0% (1/10) |
|  | 2 |  | 100% (3/3) |  | 66.7% (2/3) | 33.3% (1/3) |
| *S. aureus* | 1 | 30.0% (30/100) | 70.0% (70/100) | 30.0% (30/100) | 24.0% (24/100) | 16.0% (16/100) |
|  | 2 | 24.1% (7/29) | 75.9% (22/29) | 27.6% (8/29) | 24.1% (7/29) | 24.1% (7/29) |
| *S. oralis* | 1 | 17.3% (35/202) | 82.7% (167/202) | 31.2% (63/202) | 23.3% (47/202) | 28.2% (57/202) |
|  | 2 | 21.2% (21/99) | 78.8% (78/99) | 26.3% (26/99) | 23.2% (23/99) | 29.3% (29/99) |
| *S. pyrogenes* | 1 | 10.8% (4/37) | 89.2% (33/37) | 8.1% (3/37) | 45.9% (17/37) | 35.1% (13/37) |
|  | 2 |  | 100% (7/7) | 28.6% (2/7) | 28.6% (2/7) | 42.9% (3/7) |
| **^*^**P denotes the study Period. **^†^**n is the number of isolates in each category for each period and N is the total isolates identified as each serotype/group. The rank was determined according to colonization density. NTHI is non-typeable *Haemophilus influenzae* | | | | | | |
