# Supplementary material for: Streptococcus pneumoniae and other bacterial nasopharyngeal colonization seven years post-introduction of 13-valent pneumococcal conjugate vaccine in South African children
Source: Int J Infect Dis. 2023 Sep;134:45–52. doi: 10.1016/j.ijid.2023.05.016 (PMC10404162; doi:10.1016/j.ijid.2023.05.016)
Supplement: Supplementary file 15 [file mmc15.docx]

***Streptococcus pneumoniae* and other bacterial nasopharyngeal colonisation eight years post-introduction of 13-valent pneumococcal conjugate vaccine in South African children.**

**Sarah L. Downs^1, 2^ , Courtney P. Olwagen^1, 2^ , Lara Van Der Merwe^1, 2^, Susan Nzenze^1,3^, Marta C. Nunes^1, 2^ and Shabir A. Madhi^1, 2, 4^**

^1^ South Africa Medical Research Council Vaccines and Infectious Diseases Analytics Research Unit, University of the Witwatersrand, Faculty of Health Science, Johannesburg, South Africa

^2^ Department of Science/ National Research Foundation: Vaccine Preventable Diseases, University of the Witwatersrand, Faculty of Health Science, Johannesburg, South Africa
^3^ Division of Public Health Surveillance and Response, National Institute for Communicable Diseases of the National Health Laboratory Service; Johannesburg, South Africa

^4^ Infectious Diseases and Oncology Research Institute, University of the Witwatersrand, Faculty of Health Science, Johannesburg, South Africa.
Corresponding author: Shabir A. Madhi, Shabir.Madhi@wits.ac.za **Methods:**This study was conducted through the South African Medical Research Council and University of the Witwatersrand Vaccines and Infectious Diseases Analytics Research Unit (SAMRC/Wits-VIDA). SAMRC/Wits-VIDA is located at Chris Hani Baragwanath Academic Hospital (CHBAH) in Soweto, a settlement/township southwest of Johannesburg, Gauteng, SA. Soweto is one of the largest and oldest ‘urban’ townships within South Africa covering 200 Km^2^, with a population of 1 271 628 (6 357.29 per km²) in 355 331 (1776.42 per km²) households ^(1)^. A sample size of 408 would allow us to detect an 80%-90% individual reduction in the most prevalent PCV13-serotypes in 2009 (19F; 6B; 6A; 23F; 19A and 14) among the ≤5-year-olds.

We used WHO EPI (Expanded Program on Immunisation) two-stage systematic random sampling probability proportional to size cluster survey method (PPS) to select households (https://www.who.int/publications/i/item/WHO-IVB-18.09) from the population census data collected by Statistics South Africa (STATSSA, 2011). The Soweto community structure being diverse, ranging from high-density informal settlements to suburban neighbourhoods, was accounted for by including sociodemographic strata (informal settlements; high-density mixed-income housing; middle-class suburbs, and mixed high-density and informal settlements) in the selection process. The first stage involved categorizing enumeration areas (EAs) into the four sociodemographic strata. Subsequently, 45 EAs were selected as clusters. The second stage involved random selection of co-ordinates for ten dwelling units (DU; households) in each of the 45 EAs. DUs were first approached and if they were not eligible (no child 0-59 months in the household) the next nearest front door was approached. Households were approached once in traditional hours (Monday to Friday, 09:00-17:00) and if no one was home, they were re-approached outside of traditional hours (Saturday, 09:00-17:00), and if the second visit was unsuccessful the DU was replaced with the next nearest front door. All age-eligible children within the household were sampled. Children were excluded if they had a febrile illness (fever ≥37.5℃), used antibiotics in the three weeks before sampling, the primary caregiver was unwilling to discuss their own or their child’s HIV status (caregiver unwilling to undergo an HIV rapid test, or provide documented HIV status within 6 months of the sampling date), or if the caregiver did not provide informed consent for their child to participate.

On the day of NP sampling, information was collected about the child’s health (respiratory symptoms, fever, HIV-status and treatment, antibiotic treatment, cotrimoxazole prophylaxis, TB exposure and/or treatment, hospitalisation within three months, and medical conditions), and other co-variates (daytime social contact and breastfeeding status). When possible, the child’s HIV and PCV immunisation status was transcribed from the child’s health records, ie: Road to Health Card; RTHC. Where the RTHC was unavailable, the child’s immunisation and HIV status information were collected verbally from the caregiver.

**References:**

1. STATSSA. Census 2011 Statistical release – P0301.4 / Statistics South Africa. Pretoria: Statistics South Africa, 2012 2012.
